# TMT-Based Quantitative Proteomic Analysis Reveals the Physiological Regulatory Networks of Embryo Dehydration Protection in Lotus (*Nelumbo nucifera*)

**DOI:** 10.3389/fpls.2021.792057

**Published:** 2021-12-17

**Authors:** Di Zhang, Tao Liu, Jiangyuan Sheng, Shan Lv, Li Ren

**Affiliations:** ^1^School of Design, Shanghai Jiao Tong University, Shanghai, China; ^2^Institute for Agri-Food Standards and Testing Technology, Shanghai Academy of Agricultural Sciences, Shanghai, China

**Keywords:** *Nelumbo nucifera*, seed embryo, dehydration, desiccation tolerance, abiotic stress, TMT proteomics, parallel reaction monitoring

## Abstract

Lotus is an aquatic plant that is sensitive to water loss, but its seeds are longevous after seed embryo dehydration and maturation. The great difference between the responses of vegetative organs and seeds to dehydration is related to the special protective mechanism in embryos. In this study, tandem mass tags (TMT)-labeled proteomics and parallel reaction monitoring (PRM) technologies were used to obtain novel insights into the physiological regulatory networks during lotus seed dehydration process. Totally, 60,266 secondary spectra and 32,093 unique peptides were detected. A total of 5,477 proteins and 815 differentially expressed proteins (DEPs) were identified based on TMT data. Of these, 582 DEPs were continuously downregulated and 228 proteins were significantly up-regulated during the whole dehydration process. Bioinformatics and protein-protein interaction network analyses indicated that carbohydrate metabolism (including glycolysis/gluconeogenesis, galactose, starch and sucrose metabolism, pentose phosphate pathway, and cell wall organization), protein processing in ER, DNA repair, and antioxidative events had positive responses to lotus embryo dehydration. On the contrary, energy metabolism (metabolic pathway, photosynthesis, pyruvate metabolism, fatty acid biosynthesis) and secondary metabolism (terpenoid backbone, steroid, flavonoid biosynthesis) gradually become static status during lotus embryo water loss and maturation. Furthermore, non-enzymatic antioxidants and pentose phosphate pathway play major roles in antioxidant protection during dehydration process in lotus embryo. Abscisic acid (ABA) signaling and the accumulation of oligosaccharides, late embryogenesis abundant proteins, and heat shock proteins may be the key factors to ensure the continuous dehydration and storage tolerance of lotus seed embryo. Stress physiology detection showed that H_2_O_2_ was the main reactive oxygen species (ROS) component inducing oxidative stress damage, and glutathione and vitamin E acted as the major antioxidant to maintain the REDOX balance of lotus embryo during the dehydration process. These results provide new insights to reveal the physiological regulatory networks of the protective mechanism of embryo dehydration in lotus.

## Introduction

Lotus (*Nelumbo nucifera* Gaertn.), an ancient aquatic eudicot, belongs to the family Nelumbonaceae, which comprises only two species, *N. nucifera* and *N. lutea*, named Chinese lotus and American lotus, respectively (Shen-Miller, 2002; Shen-Miller et al., 2013). Lotus is among the top ten famous flowers in China, and it has been cultivated in China as vegetables and medicinal plants for over 7,000 years (Guo, 2009). Lotus has adapted to aquatic environments and plays an important role in wetland preservation and ecological restoration worldwide (Ming et al., 2013). The seed plays an important role in plant life cycle. It stores genetic information and nutrients to guarantee the reproduction of the next generation (Wang et al., 2016). Lotus seeds are black, hard, and ovoid. In its late immature stages, the seed is covered in a soft green husk containing a moist and soft endosperm and the developing embryo. When the seed reaches maturity, the husk turns dark brown and hardens, and both the endosperm and embryo become considerably dry (Moro et al., 2015). Lotus seeds have the characteristics of longevity and extremely durable storage, known as the “millennium ancient lotus seed.” At the beginning of the twentieth century, some ancient lotus seeds were excavated from peat layer in Liaoning province of China, which can still germinate and grow normally. According to ^14^C isotope detection, these seeds had a lifespan of more than 1,300 years, being one of the longest-lived seeds (Shen-Miller, 2002). The great difference between the responses of vegetative organs and seeds to dehydration is related to the special protective mechanism in embryos.

Seed longevity varies greatly between different plants, which can be as short as a few hours or as long as thousands of years (Ming et al., 2013). The difference in seed life span depends on genetic and environmental factors. Seed coat and pericarp have sealing function and play an important role in protecting seed vigor; however, lotus seeds removed pericarp can maintain 100% germination rate and develop into normal seedlings after 100°C treatment for 24 h (Huang et al., 2003), suggesting that the special anti-stress and protection mechanism of lotus embryo is an important internal cause to ensure the longevity of lotus seed. Lotus seeds mainly accumulate starch, which accounts for about 60% of its total dry weight. It also accumulates about 8% protein in immature seeds and as high as 24% in the mature desiccated seeds (Wang et al., 2016). Metabolomic and proteomic profiles revealed a highly significant metabolic switch at 15 days after pollination (DAP) going through a transition of metabolically highly active tissue to the preparation of storage tissue (Wang et al., 2016). Seed life span is a complex biological process from quantitative change to qualitative change. Seed formation is classically divided into two major phases: early embryogenesis during which the pattern and morphology of the embryo are established, and late embryogenesis during which seeds accumulate storage products, acquire desiccation tolerance, and finally fall in dormancy (Delseny et al., 2001). A series of complex and multifaceted responses are involved in desiccation tolerance in plants, including the structural or component alteration of cell wall, organelles, or organs, induction of the repair system, removal of free radicals, and accumulation of macromolecules. During seed desiccation, the accumulation of macromolecules such as oligosaccharides and proteins greatly increases cytoplasmic viscosity and usually causes the formation of bioglasses. Thus, bioglasses have been suggested to provide intracellular protection against the denaturation of large molecules to stabilize plasma membranes (Burke, 1986; Shih et al., 2008). On the molecular level, a number of mechanisms influencing seed survival in the dry state have been discovered in many plants. These mechanisms include the synthesis of protective molecules, such as non-reducing sugars including raffinose, stachyose, and cyclitols (Hoekstra et al., 2001; Verdier et al., 2013), late embryogenesis abundant (LEA) proteins (Hundertmark et al., 2011; Chatelain et al., 2012), heat shock proteins (HSPs) (Prieto-Dapena et al., 2006), various other stress proteins (Sugliani et al., 2009), and a set of antioxidant including glutathione (Kranner et al., 2006), tocopherols (Sattler et al., 2004), and flavonoids (Debeaujon et al., 2000). Interestingly, the phytohormone abscisic acid (ABA) is a central regulator of plant development and responses to environmental stresses. ABA controls seed developmental processes, including accumulation of food reserves, the acquisition of dormancy, and desiccation tolerance (Dekkers et al., 2015).

The genome of lotus has been sequenced and annotated recently (Ming et al., 2013; Wang et al., 2013), which has provided ample information for genomics and proteomics research of this aquatic species. Previous studies on lotus seed were mainly focused on the identification of its nutritional constituents, medicinal components, and biosynthesis regulation (Mukherjee et al., 2010; Wang et al., 2016). However, there are few reports on the systematic identification and characterization of dehydration tolerance and dehydration stress-related proteins in lotus seeds. Dehydration tolerance research has diverse applications, such as improving drought tolerance of crop species, optimizing germplasm resources, and stabilizing biomolecules and eukaryotic cells. In this study, TMT-labeled proteomics and PRM technologies were used to obtain novel insights into the biological network that regulates lotus seed dehydration tolerance and dehydration stress and laid a theoretical foundation for revealing the mystery of lotus seed longevity.

## Materials and Methods

### Plant Materials

Lotus (*N. nucifera* “TaiKong 36”) seeds were obtained from a cultivation pond under natural conditions in Shanghai Jiao Tong University, Shanghai, during the summer of 2018. Lotus plants are manually pollinated every morning and recorded the time from June to July. The developing seeds were collected at 15, 18, 21, 24, 27, 30, 40, and 50 DAP. The seed coat and cotyledon tissue were removed carefully by sharp shears, and 0.2 g of lotus embryos were collected and stored in a centrifugal tube. The collected lotus embryo samples were immediately frozen in liquid nitrogen and stored at −80°C for further proteomic analysis and physiological measurements.

### Detection of Physiological Indices

For relative water content (RWC) detection, the embryo at different developmental stages were weighted for fresh weight (FW) and then dried at 70°C for constant weight (DW). The RWC was calculated using the following formula: RWC = (FW–DW)/FW ^*^ 100%. Five biological replicates per seed developmental stage were detected. A DDS-307 conductivity meter (INESA, Shanghai, China) was used to determine the relative electrical conductivity (REC) as described by Yang et al. (2019). Soluble sugar and soluble protein content were used with anthracene ketone-sulfuric acid and Coomassie bright blue (G250) method to detect the absorbance at 620 and 595 nm, respectively. Catalase (CAT), superoxide dismutase (SOD) and peroxidase (POD) activities, superoxide anion (${\text{O}}_{2}^{-}$) inhibition activity, H_2_O_2_ content, hydroxyl radical (OH·) inhibition activity, ascorbic acid (AsA), glutathione (GSH), tocopherol (vitamin E, V_E_), and malondialdehyde (MDA) contents were detected using biological assay kits (Nanjing Jiancheng Bioengineering Institute, China) following the manufacturer's instructions. ABA concentration was measured using a phytohormone ELISA Kit (Shanghai Enzyme-Linked Biotechnology Co., Ltd.) following the manufacturer's protocol. Three biological replicates were detected.

### Protein Extraction

The protein extraction was performed according to Bo's et al. (2020) method with minor modifications. Frozen lotus embryos were mixed the steel beads and ground at the power of 60 Hz for 2 min. The samples were then supplemented with 1 ml of extraction buffer and mixed with Tris-phenol buffer for 30 min at 4°C. Next, the mixtures were centrifuged at 7,100 *g* for 10 min at 4°C and the phenol supernatants were collected. The supernatants were added to 5 times the volumes of 0.1 M cold ammonium acetate-methanol buffer and precipitated at −20°C overnight. After precipitation, the samples were centrifuged at 12,000 *g* for 10 min and the precipitation was collected. Then, the precipitation was washed by cold methanol and centrifuged at 12,000 *g* for 10 min at 4°C and the precipitation was collected. Methanol was then replaced by acetone and the wash stem was repeated twice to remove methanol contamination. Afterward, the samples were centrifuged at 12,000 *g* for 10 min at 4°C to collect precipitation and dried at room temperature for 3 min and then dissolved in lysis buffer for 3 h. Finally, the samples were centrifuged at 12,000 *g* for 10 min to collect supernatants. The supernatants were centrifuged again to remove precipitation completely. Protein concentration was determined by a BCA assay kit (Sangon Biotech, Shanghai, China) and aliquoted to store at −80°C.

### Protein Digestion and TMT Labeling

Protein digestion was performed according to the filter-aided sample preparation (FASP) method (Wiśniewski et al., 2009). Briefly, the proteins were digested with trypsin (enzymes/substrate ratio 1:50) overnight after reductively alkylation with 10 mM dithiothreitol (DTT) (1 h, 60°C) and 50 mM iodoacetamide (IAA) (40 min, room temperature). The digested peptides were lyophilized and resolved in 200 mM triethylamine buffer (TEAB). Finally, the lyophilized samples were labeled using the 10 plex TMT reagent kit (Thermo scientific, USA) following the manufacturer's instructions. Briefly, the tryptic peptides (100 μg each) were labeled with TMT 10-plex with 126-tag (21d-1), 127N-tag (21d-2), 127C-tag (21d-3), 128N-tag (27d-1), 128C-tag (27d-2), 129N-tag (27d-3), 129C-tag (40d-1), 130N-tag (40d-2), 130C-tag (40d-3), and 131-tag (MIX). Proteomics platform was provided by Shanghai Luming Biotech. Co., Ltd. (Shanghai, China).

### Mass Spectrometry Analyses

Mass Spectrometry (MS) detection was carried out as previously described (Gong et al., 2021). Reversed phase (RP) separation was performed on a 1,100 HPLC System (Agilent) using an Agilent Zorbax Extend RP column (5 μm, 150 × 2.1 mm). Mobile phases A (2% acetonitrile) and B (98% acetonitrile) were used for RP gradient. The solvent gradient was set as follows: 0–8 min, 98% A; 8.00–8.01 min, 98–95% A; 8.01–48 min, 95–75% A; 60–60.01 min, 60–10% A; 60.01–70 min, 10% A; 70–70.01 min, 10–98% A; 70.01–75 min, 98% A. Tryptic peptides were separated at a fluent flow rate of 300 μl/min and monitored at 210 and 280 nm. Dried samples were harvested from 8 to 50 min and elution buffer was collected every minute. The separated peptides were lyophilized for MS detection.

Mass spectrometry analyses were performed by a Q-Exactive mass spectrometer (Thermo, USA) equipped with a Nanospray Flex source (Thermo, USA). Samples were loaded and separated by a C18 column (15 cm × 75 μm) on an EASY-nLC^TM^ 1,200 system (Thermo, USA). The flow rate was 300 nl/min and the linear gradient was 60 min. Afterward, intact peptides were detected in the Orbitrap at a resolution of 70,000 with an automatic gain control (AGC) target of 1e6. The 10 most abundant precursor ions from the survey scan (300–1,600 *m/z*) were selected for higher-energy collisional dissociation (HCD) fragmentation at a normalized collision energy of 32. The resulting fragments were analyzed with the Orbitrap at a resolution of 35,000 with an AGC target of 2e5, maximum inject time of 80 ms, and dynamic exclusion duration of 30.0 s.

### Protein Identification and Bioinformatics Analyses

The raw MS/MS data were processed using Proteome Discoverer (Version 2.2) software and tandem mass spectra were searched against the Uniprot-proteome_UP000189703-*Nelumbo nucifera* (Sacred lotus) (Strain cv. China Antique) database. Peptides with FDR <1% are considered to be effective. Credible proteins are selected based on the criteria of unique peptide ≥1 and Score Sequest >0, and the blank value is removed.

*T*-test was performed on three biological replicates in each group to calculate the statistical significance of all identified proteins between the comparison groups, and the average of the three biological replicates was used as the final protein expression rate. In this study, differentially expressed proteins (DEPs) were defined by the fold change criteria (FC) ≥ 1.5 or ≤ 0.67 (*p* < 0.05). Expression pattern clustering of all identified DEPs was performed using Genesis 1.8.1 software. Gene ontology (GO) function and Kyoto encyclopedia of genes and genomes (KEGG) pathway enrichment annotation were analyzed using the Omicsbean platform (www.omicsbean.cn). The protein interaction regulatory network was constructed by using STRING website (https://string-db.org).

### PRM Analysis

Parallel Reaction Monitoring (PRM) assay was performed as previously described with minor modifications (Bo et al., 2020; Gong et al., 2021). Eighteen key candidate DEPs were selected from the TMT data. PRM was used to quantify the expression, which was performed by Shanghai Ouyi Biological Co., Ltd. (Shanghai, China). The protein exaction was the same as the above procedure. The digested peptides were submitted to the PRM analysis. Three replicates for each sample were evaluated. The signature peptides of the target proteins were defined by the TMT dataset, and only the unique peptide sequence was selected for PRM analysis. In brief, the tryptic peptides were mixed in the solvent A and eluted in a reversed phase analytical column using the gradient solvent B (8–25% over 60 min, 25–45% over 20 min, 45–100% over 1 min, and 100% over the last 10 min) at a rate of 300 nl/min. The peptides measured were analyzed with a Q Exactive HF Orbitrap mass spectrometer (ThermoFisher Scientific, Waltham, MA, USA). The full MS scans (350–16,500 m/z) were obtained at a resolution of 1,200,000 using AGC of 3e6 and a highest injection time (MIT) of 100 ms. A data independent protocol (one MS scan followed by 20 MS/MS scans) was used for the MS/MS scans with the following parameters: resolution, 30,000; NEC, 27; AGC, 2E5; MIT, 80 ms; and insulation window, 1.4 m/z. The PRM data was analyzed using Skyline 3.6. The results were quantified for every peptide, and the DEPs detected were screened and compared with the MS data derived from the TMT.

### Statistical Analysis

All experiments were repeated at least three times, and statistical analysis was performed using SAS 9.1.3 software and one-way ANOVA followed by least significant difference (LSD) multiple range tests (*p* < 0.05). The correlation analysis of oxidative physiological parameters under stresses was performed using SAS 9.1.3 software. *p* < 0.05 was considered significant.

## Results

### Development and Dehydration Characteristics of Lotus Seed Embryos

**Figure 1:**
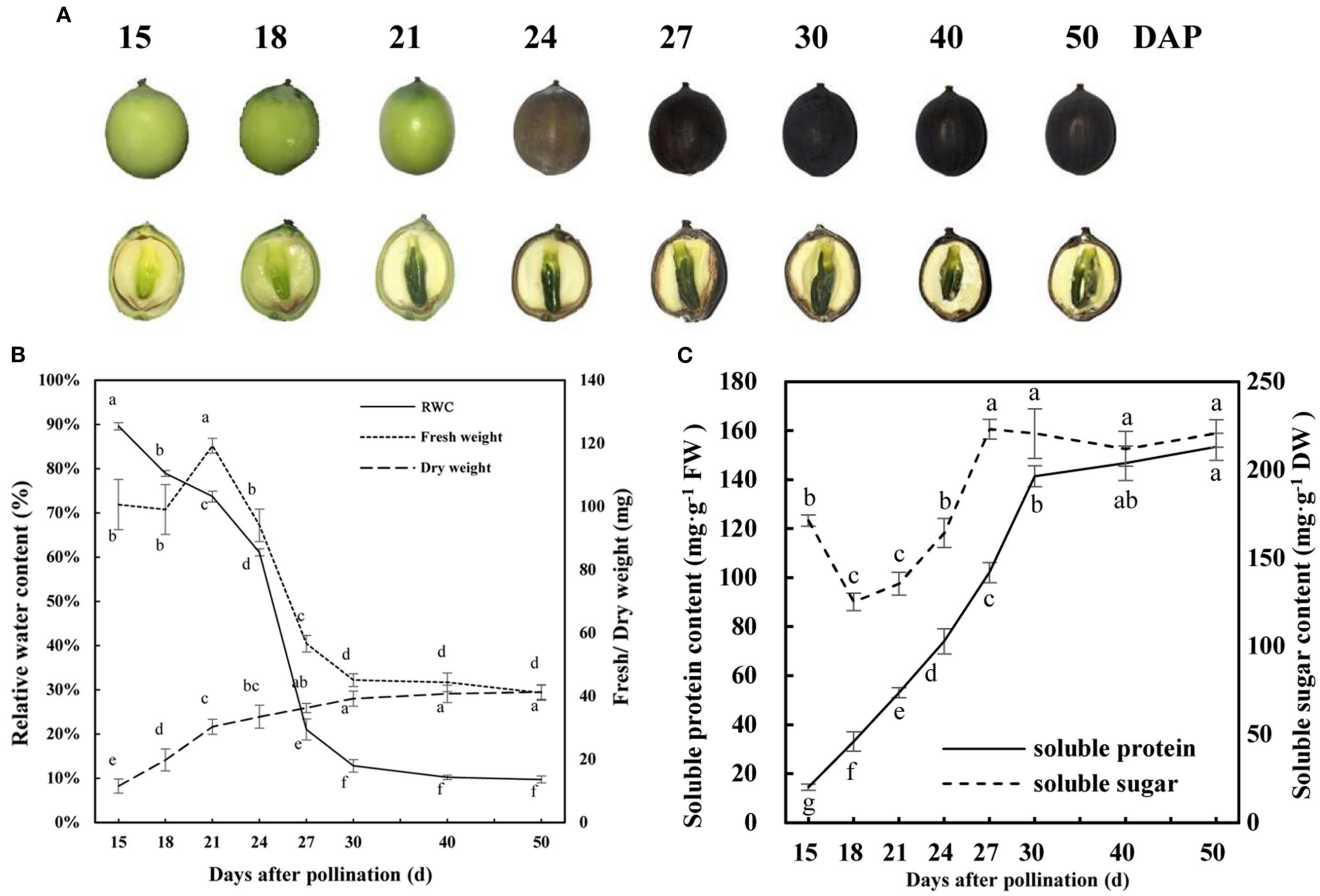
Development and dehydration characteristics of lotus seed embryos. **(A)** Morphological traits of developing lotus seed; **(B)** Fresh/dry weight and relative water content of developing lotus seed embryo; **(C)** Soluble protein and sugar contents of developing lotus seed embryo. Lower-case letters indicate that the indexes have significant differences between different developmental stages; *p* < 0.05.

The whole process of the development and maturation of lotus seeds needs about 40 d, during which the morphological and color characteristics of embryo and pericarp changed clearly (Figure 1A). In the first stage, the embryo mainly undergoes embryogenesis and development. The embryo reached morphological maturity during 18–21 DAP, and embryo and pericarp appeared bright green color, the fresh weight of embryo reached the maximum, and dry matter accumulated rapidly during this stage (Figure 1). In the second stage, lotus seeds enter the dehydrated mature process from 21 to 50 DAP, the embryo and pericarp gradually shrunk in shape, and the color gradually became dark brown (Figure 1A). The RWC (dropped from 73.7 to 21.0%) and fresh weight (dropped from 119.3 to 56.6 mg) of the embryo decreased dramatically, and dry matter accumulated slowly during 21–27 DAP (Figure 1B). The soluble sugar and soluble protein contents increased significantly during the rapid dehydration stage (18–27 DAP) (Figure 1C). Then, the RWC, fresh weight, dry weight, and soluble sugar and proteins of lotus embryos enter the steady phase during 30–50 DAP (Figures 1B,C). These results indicated that the lotus embryo reached morphological maturity, rapid dehydration, and dehydration maturity at 21, 27, and 40 DAP, respectively. Accordingly, embryos from these three time points were used for subsequent proteomic studies.

### TMT Data and DEP Identification

**Figure 2:**
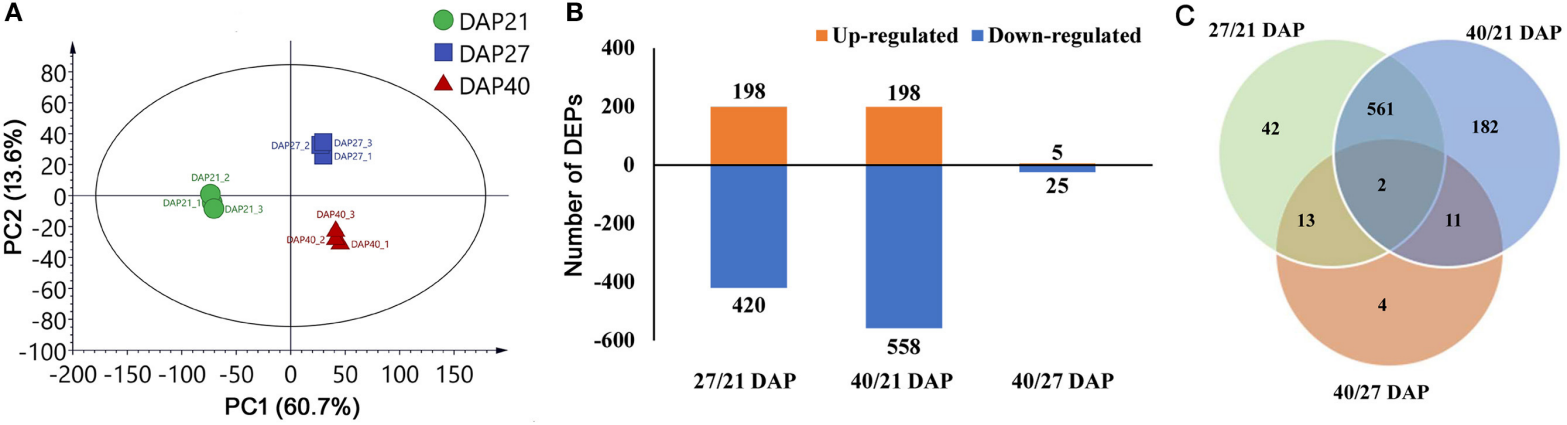
Statistic of differentially expressed proteins (DEPs) of lotus embryos during seed dehydration maturity. **(A)** Principal components analysis of each sample; **(B)** Statistics of up and down-regulated DEPs; **(C)** Veen diagram analysis of DEPs among different groups.

Proteome analysis of fresh lotus embryos at the three dehydration stages (21, 27, and 40 DAP) was performed by TMT labeling technology. Totally, 60,266 secondary spectra and 32,093 unique peptides were detected. After removing repetitive proteins, 5,477 proteins were identified based on TMT data. The statistical information of identified proteins is shown in Supplementary Table 1, including the molecular weight, peptide sequence length, distribution of peptide number, and protein quantification. PCA analysis was performed to evaluate the test samples, and the results showed that the different groups of lotus embryos were well-distinguished, and three repeated samples of each group were aggregated together. The first two components of PCA accounted for more than 70 % of the total variance, separating the total proteins from the different groups (Figure 2A). A total of 815 protein abundance changed significantly between the three test groups. Of these, 198 (32%) and 198 (26%) proteins increased in abundance, while 420 (68%) and 558 (74%) proteins decreased in abundance in the 27 DAP and 40 DAP groups, respectively (Figure 2B). Only 30 proteins abundance changed significantly between 27 DAP and 40 DAP groups (Figure 2C). Quantitative and functional annotation information of all DEPs is shown in Supplementary Table 2.

### Bioinformatics Analysis of the DEGs

**Figure 3:**
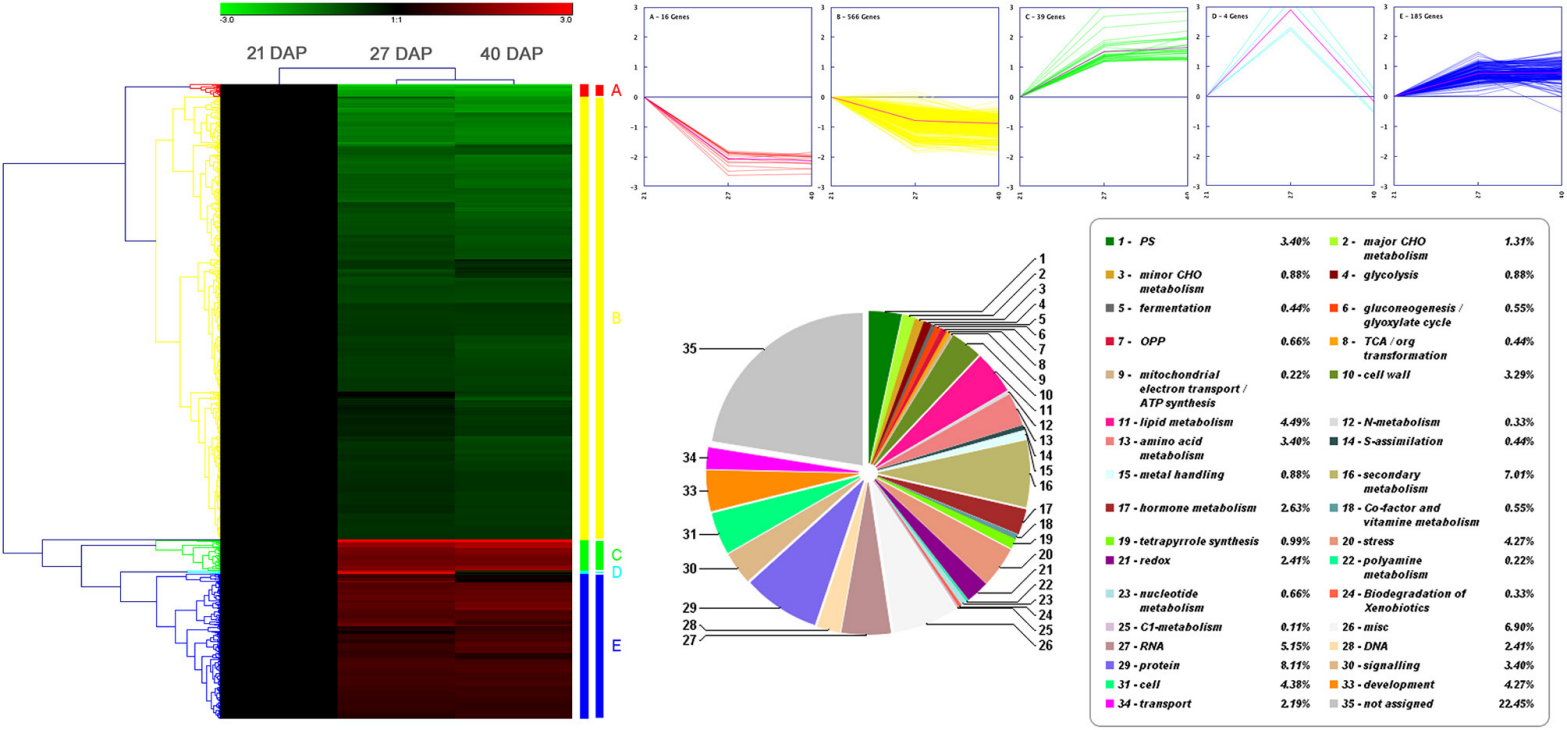
Hierarchical clustering and protein functional classification analysis of 815 DEPs in lotus embryos during seed dehydration maturity.

The 815 DEPs identified from lotus embryos during seed dehydration maturity were conducted using hierarchical clustering and protein functional classification analysis (Figure 3). All DEPs were classified into 5 main expression profiles. Cluster A (contains 16 proteins) and cluster B (566 proteins) contained DEPs that were continuously downregulated, and cluster C (39 proteins) and cluster E (185 proteins) were gradually up-regulated during the seed dehydration process. Cluster D only contains 4 proteins that had a specific up-regulated expression at the rapid dehydration stage (27 DAP), including dehydrin RAB18 (A0A1U7ZRR9), digalactosyldiacylglycerol synthase (A0A1U7ZHL0), protein TORNADO (A0A1U7ZLU9), and an uncharacterized protein (A0A1U8B9X3). Protein functional classification analysis showed that 815 DEPs encoded proteins that were mainly associated with photosynthesis, lipid metabolism, amino acid metabolism, hormone metabolism, RNA, protein processing, cell wall, secondary metabolism, stress, and development (Figure 3).

Pathway and GO annotation and significant enrichment methods were used for understanding the seed embryo dehydration events and associated downstream biological processes regulated by the DEPs. According to the results of clustering analysis, the DEPs were divided into up-regulated expression groups (clusters C and E) and downregulated expression groups (clusters A and B), which contained 224 and 582 proteins, respectively. A total of 224 up-regulated DEPs were annotated to GO terms (Supplementary Figure 1A), and the main categories in biological process (BP) include response to stress (74, 37.2%), response to chemical (64, 32.2%), and response to abiotic stimulus (59, 29.6%); the prominent cell component (CC) categories were cell part (152, 76.4%) and intracellular part (135, 67.8%); and the most significant categories in molecular function (MF) were carbon-carbon lyase activity (6, 3.0%) and sulfur compound binding (4, 2.0%). A total of 582 downregulated DEPs were performed with GO annotation (Supplementary Figure 1B), and the main categories in BP were single-organism process (395, 75.7%) and single-organism cellular process (339, 64.8%); the prominent CC categories were cell part (461, 88.1%), intracellular part (422, 80.7%) and chloroplast; and the most significant categories in MF were protein binding (143, 27.3%) and nucleoside binding (72, 13.8%). Additionally, AgriGO significant enrichment analysis showed that up-regulated DEPs were significantly enriched in “carbohydrate metabolism process,” “catalytic activity,” and “RNA banding” GO terms, and downregulated DEPs were significantly enriched in “amino acid and derivative metabolic process,” “carbohydrate metabolism process,” “lipid metabolic process,” “generation of precursor metabolites and energy,” “photosynthesis,” “cytoskeleton,” “Golgi apparatus,” “endoplasmic reticulum,” and “peroxisome” GO terms (Supplementary Figure 2). KEGG pathway annotation analysis showed that the 815 DEPs were significantly enriched in “pyruvate metabolism,” “glycolysis/gluconeogenesis,” “pentose phosphate pathway,” “fatty acid biosynthesis and metabolism,” “biosynthesis of amino acids,” “amino sugar and nucleotide sugar metabolism,” “glutathione metabolism,” “terpenoid backbone biosynthesis,” “steroid biosynthesis,” “flavonoid biosynthesis,” “photosynthesis,” “biosynthesis of secondary metabolites,” and “metabolic pathway” (Supplementary Figure 3). Furthermore, the up-regulated DEPs were mainly enriched in “galactose metabolism,” “pentose phosphate pathway,” “glycolysis/gluconeogenesis,” “protein processing in endoplasmic reticulum,” and “glutathione metabolism;” and the downregulated DEPs were significantly enriched in “metabolism pathway,” “biosynthesis of secondary metabolites,” “pyruvate metabolism,” “photosynthesis,” and “fatty acid biosynthesis and metabolism” (Supplementary Figure 4). The above results suggested that environmental stimuli, stress response, energy metabolism, and protein processing in ER-related biological process and pathway are vigorous during lotus embryo continuous dehydration and maturation. Nevertheless, the cell metabolism, biosynthesis of secondary metabolites, photosynthesis, amino acids biosynthesis, and fatty acid metabolism are distinctly decreased or inhibited in this water loss process.

### Protein-Protein Interaction Networks Analysis of DEPs

**Figure 4:**
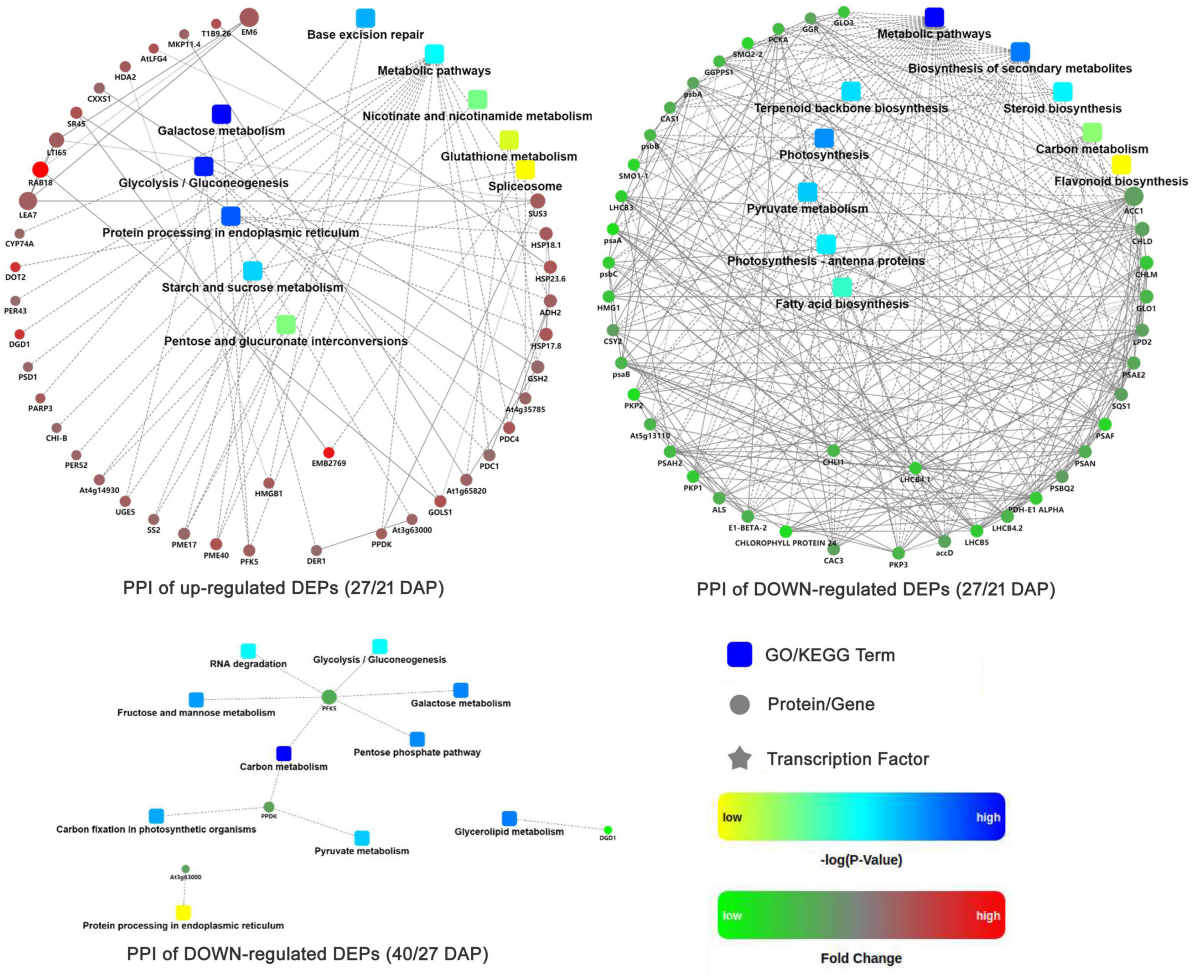
Protein-protein interaction networks of DEPs at different seed dehydration stage of lotus embryos.

**Figure 5:**
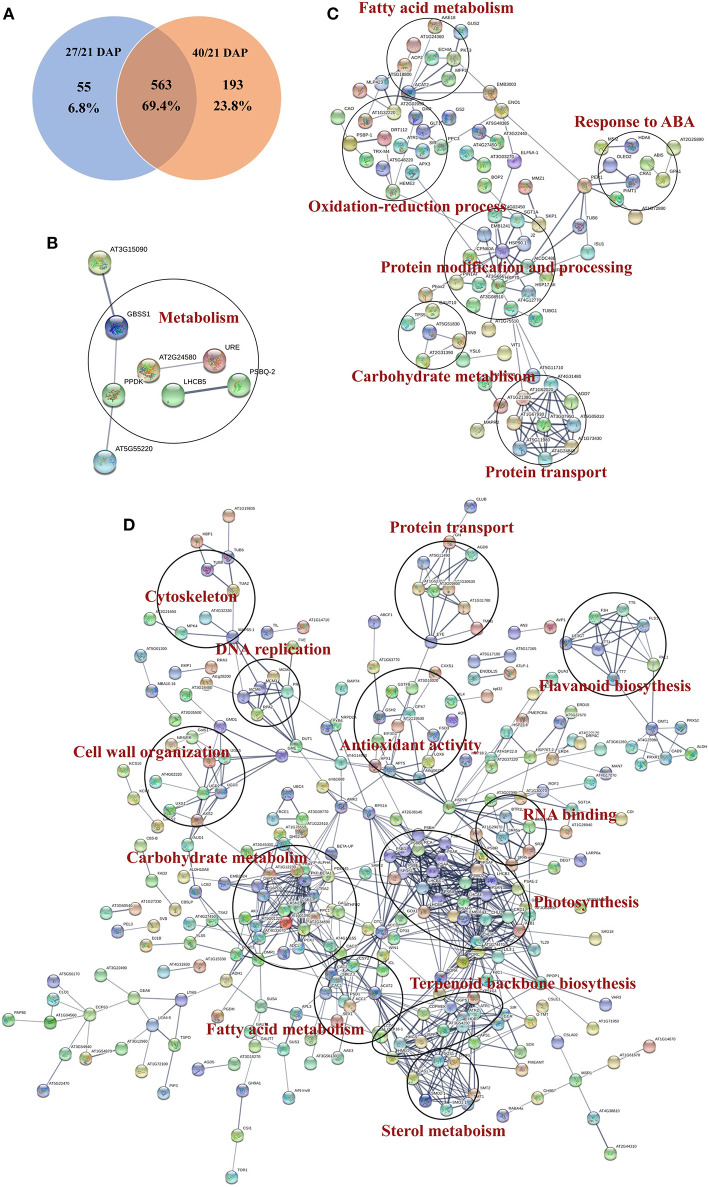
Functional protein association networks analysis of DEGs from tandem mass tags (TMT) proteomics data. **(A)** Venn diagram; **(B)** Interaction network of common DEPs; **(C)** Interaction network of DEPs in 27 days after pollination (DAP); **(D)** Interaction network of specific DEPs in 40 DAP.

Protein-protein interaction (PPI) networks analysis were performed based on the DEPs during different seed dehydration stage, i.e., 618 DEPs from rapid dehydration stage (27/21 DAP) and 30 DEPs from terminal dehydration stage (40/27 DAP). In the rapid dehydration stage, up-regulated DEPs interact with each other and participate in “galactose metabolism,” “glycolysis/gluconeogenesis,” “protein processing in ER,” “starch and sucrose metabolism,” and “base excision repair” GO/KEGG terms, and dehydrin Rab18-like (A0A1U7ZRR9), STAR-1 Family DOT2 (A0A1U8A3P7), and digalactosyldiacylglycerol synthase (A0A1U7ZHL0) have the highest fold change of protein abundance in this network (Figure 4). PPI network of downregulated DEPs (27/21 DAP) mainly include “metabolic pathway,” “biosynthesis of secondary metabolites, “photosynthesis,” “pyruvate metabolism,” “terpenoid backbone biosynthesis,” and “fatty acid biosynthesis” (Figure 4). In the terminal dehydration stage (40/27 DAP), only downregulated DEPs can construct PPI networks, and annotated proteins were involved in “carbon metabolism,” “galactose metabolism,” “pyruvate metabolism,” “glycerolipid metabolism,” and “protein processing in ER.” Furthermore, according to the DEP Venn diagram of 27/21 DAP and 40/21 DAP, 563 (69.4%) proteins were overlapped in lotus embryos at 27 DAP and 40 DAP, while 55 (6.8%) and 193 (23.8%) DEPs were unique at 27 DAP and 40 DAP, respectively (Figure 5A). PPI networks calculated by the STRING database showed that 27 DAP-specific DEPs were involved in the metabolism pathway (Figure 5B) while 193 specific DEPs of the 40 DAP group were related to 6 biological processes including fatty acid metabolism, oxidation-reductive process, response to ABA, carbohydrate metabolism, protein modification and processing, and protein transport (Figure 5C). The overlapped DEPs were mainly involved in 12 biological processes, such as cell wall organization, carbohydrate metabolism, RNA binding, carbohydrate and fatty acid metabolism, and antioxidant activities (Figure 5D). These PPI networks results indicated that carbohydrate metabolism (including glycolysis/gluconeogenesis, galactose, starch and sucrose metabolism, pentose phosphate pathway, and cell wall organization), protein processing in ER, DNA repair, and antioxidative events have a positive response to lotus embryo dehydration and maturation, and ABA signal and dehydrin Rab18 protein may play an important role in this process. On the contrary, the energy metabolism (metabolic pathway, photosynthesis, pyruvate metabolism, and fatty acid biosynthesis) and secondary metabolism (including terpenoid backbone, steroid, and flavonoid biosynthesis) gradually become static status during lotus embryo dehydration and maturation. Based on the above bioinformatics analysis, a total of 92 key DEPs were selected and shown in Supplementary Table 3. Those proteins were involved in carbohydrate and energy metabolism, redox homeostasis, stress/defense, protein modification and degradation, response to ABA signaling, and DNA repair.

### PRM Validation

To validate the TMT proteomic data, 18 candidate DEPs that were closely related to the dehydration protection event of lotus seed embryo were selected for PRM quantitative detection (Supplementary Figure 5). PRM identified 18 candidate DEPs which include pyruvate decarboxylase, glucose-6-phosphate dehydrogenase, phosphoenolpyruvate carboxylase, citrate synthase, sucrose synthase, acetyl-CoA carboxylase 1-like, glutathione S-transferase-like, L-ascorbate peroxidase, thioredoxin-like, allene oxide synthase, protein-L-isoaspartate O-methyltransferase, dehydrin Rab18-like, LEA EMB564, LEA D34-like, LEA D34, cysteine proteinase inhibitor, histone deacetylase, and DNA damage repair protein. These protein expression patterns showed similar trends between PRM and TMT quantitation results. However, the fold change value of protein abundance detected by PRM was greater than that of TMT data (Table 1). This result largely supports the reliability of the TMT data.

**Table 1:** Comparison of the quantitation results between tandem mass tags (TMT) and parallel reaction monitoring (PRM).

**Accession**	**Description**	**TMT result (FC)**		**PRM result (FC)**	
		**27/21**	**40/21**	**27/21**	**40/21**
A0A1U8AFZ9	Pyruvate decarboxylase 2	2.286	2.416	4.480	6.323
A0A1U7ZDJ8	Glucose-6-Phosphate 1-Dehydrogenase	1.517	1.553	1.733	2.727
A0A1U8BJ10	Phosphoenolpyruvate carboxylase	0.650	0.548	0.382	0.455
A0A1U8BAL0	Citrate synthase	0.652	0.576	0.343	0.489
A0A1U8APR5	Sucrose synthase	2.069	2.302	1.783	5.099
A0A1U8B0W4	Acetyl-CoA carboxylase 1-like	0.652	0.636	0.218	0.345
A0A1U8A0H7	Glutathione S-transferase-like	1.553	1.570	ns	1.750
A0A1U8Q237	L-Ascorbate peroxidase 2	0.202	0.188	0.006	0.005
A0A1U7ZHC7	Thioredoxin-like protein CXXS1	1.639	1.908	1.883	3.239
A0A1U7ZLU2	Allene oxide synthase 1	1.669	1.684	1.756	4.629
A0A1U7ZBZ6	Protein-L-Isoaspartate O- methyltransferase	ns	1.508	ns	2.376
A0A1U7ZRR9	Dehydrin Rab18-like	14.646	ns	2.044	1.761
A0A1U8ACK5	Late embryogenesis abundant protein EMB564	2.173	2.241	2.435	3.244
A0A1U8BJB0	Late embryogenesis abundant protein D-34-like	2.230	2.304	3.856	5.512
A0A1U7ZRG9	Late embryogenesis abundant protein D-34	2.203	2.338	3.658	4.603
A0A1U7YX92	Cysteine proteinase inhibitor	2.215	2.383	3.246	4.488
A0A1U8Q4V5	Histone deacetylase 2	2.110	1.858	3.030	4.213
A0A1U7ZYD0	DNA damage repair protein	1.730	1.676	1.956	3.014

*“ns” means there was no significant difference in protein abundance changes, and gray background indicates the results of PRM and TMT were different*.

### Stress Physiological Indicators Validation

**Figure 6:**
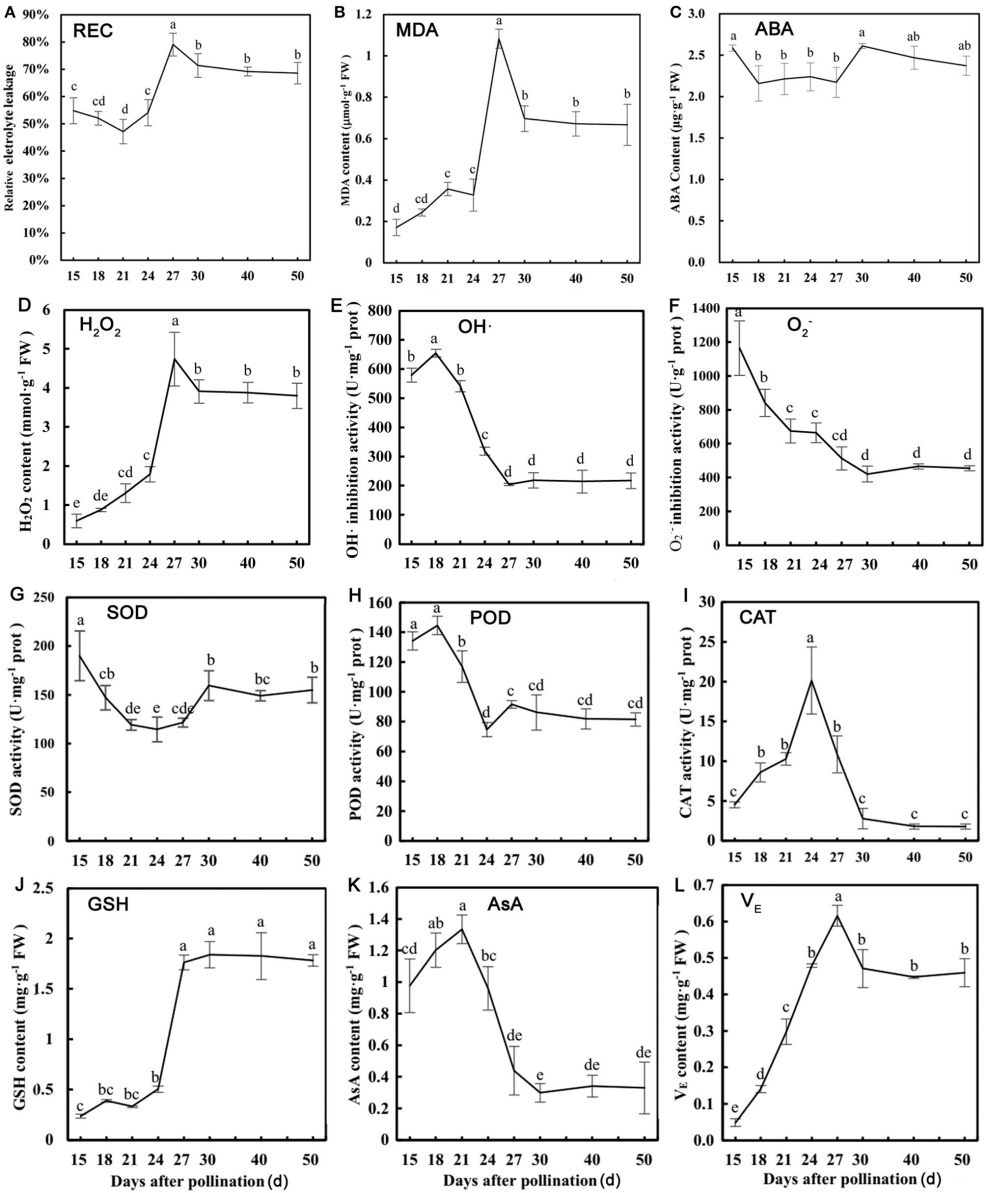
Physiological parameters detection of lotus embryos at different seed dehydration stages. **(A)** Relative electrical conductivity (REC); **(B)** malondialdehyde (MDA) content; **(C)** abscisic acid (ABA) content; **(D)** H_2_O_2_ content; **(E)** hydroxyl radical (OH·) inhibition activity; **(F)** ${\text{O}}_{2}^{-}$ inhibition activity; **(G)** superoxide dismutase (SOD) activity; **(H)** peroxidase (POD) activity; **(I)** catalase (CAT) activity; **(J)** glutathione (GSH) content; **(K)** ascorbic acid (AsA) content; **(L)** tocopherol (V_E_) content. Lower-case letters represent the significant differences (*p* < 0.05, least significant difference test) between different dehydration stages of seed embryos.

Based on the bioinformatics analysis of TMT proteomics, stress physiological indicators were examined to verify protective mechanism of dehydration in lotus seed embryos. The change of REC and MDA contents was opposite to that of RWC as their levels increased rapidly during rapid dehydration stage (Figures 6A,B). ABA content increased slightly before the rapid dehydration and dehydration maturity stages (Figure 6C). The changing trend of the above indicators suggested that ABA as an upstream signal is involved in inducing dehydration process, and serious membrane lipid peroxidation and plasma membrane damage occurred during rapid dehydration stage in lotus embryo. ${\text{O}}_{2}^{-}$ and OH· inhibition activities were significantly decreased and H_2_O_2_ levels were increased 7-fold during 18–27 DAP (Figures 6D–F), which indicated that excessive ROS components are produced during embryo dehydration process. Additionally, CAT activities were significantly increased at the early stage of dehydration and then continuously decreased. SOD and POD activities showed gradually declines in response to embryo water loss (Figures 6G–I). Meanwhile, GSH and V_E_ contents increased 7.3 and 4.5 times, respectively, from 15 to 27 DAP, and then their levels tend to stabilize. However, AsA, as a water-soluble antioxidant, was continuously decreased during embryo dehydration process (Figures 6J–L). Furthermore, the correlation analysis of stress physiological indicators showed that REC had a significant negative correlation to MDA (*p* < 0.01), POD (*p* < 0.05), H_2_O_2_ (*p* < 0.01), GSH (*p* < 0.01), and V_E_ (*p* < 0.05). REC and MDA content had a significant positive correlation to H_2_O_2_, GSH, and V_E_ (Supplementary Table 3). H_2_O_2_ levels showed a significant positive correlation to GSH and V_E_, and had a negative correlation to AsA and ${\text{O}}_{2}^{-}$ contents and OH· inhibition activities (Supplementary Table 3). These results indicated that H_2_O_2_ was the main ROS component inducing oxidative stress damage, and GSH and V_E_ acts as the major antioxidant to maintain the REDOX balance of lotus embryo during the dehydration process.

## Discussion

### Carbohydrate and Energy Metabolism During Lotus Seed Dehydration and Maturity

Glycolysis, tricarboxylic acid (TCA) cycle, and pentose phosphate pathway (PPP) are the three main pathways of carbohydrate and energy metabolism in plants. Glycolysis is a cytoplasmic pathway that breaks down glucose into two pyruvates and generates energy such as ATP and NADH. In aerobic conditions, pyruvate, through oxidative decarboxylation reaction, enters the TCA cycle that intermediates energy metabolism and participates in carbohydrates, amino acid, and fatty acids metabolism (Zhang et al., 2013). The PPP is different from the first two carbohydrate metabolism pathways, since it is no consumption and production of ATP during the reaction. The proportion of the PPP is relatively low in plant cells in normal environments, but increase when plants are under stress conditions, and the reduced state of glutathione and the redox balance of plant cells could be maintained by PPP (Kruger and von Schaewen, 2003; Scheibe, 2004; Scharte et al., 2009). The proteomics study of rice grains during development found that proteins involved in glycolysis and TCA cycle accumulated at a high level at the end of grain maturity (Lee and Koh, 2011). In wheat seeds, glycolysis and starch synthesis was significantly enhanced at the late stage of grain filling, which provided much energy for grain maturity (Zhang N. et al., 2015; Zhang et al., 2021). In this study, 16 DEPs in lotus embryo involved in the glycolysis pathway, of which 7 proteins (fructokinase, pyruvate decarboxylase, fructose-bisphosphate aldolase, etc.) were up-regulated and 9 proteins (isocitrate lyase, phosphoenolpyruvate carboxylase, phosphoenolpyruvate carboxykinase, etc.) were downregulated. However, 5 proteins involved in TCA cycle were all significantly downregulated (Supplementary Figure 6), and two significantly up-regulated proteins were found to participate in PPP, including glucose-6-phosphate dehydrogenase (G6PDH, A0A1U7ZDJ8) and 6-phosphate gluconolactonease (A0A1U7ZD76). G6PDH, as the key rate-limiting enzyme in PPP, can promote the regeneration of reduced glutathione and improve the cellular antioxidant capacity. Therefore, the carbohydrate and energy metabolism of lotus embryos decrease with dehydration and maturity, and PPP plays an important role in maintaining the redox homeostasis and alleviating stress damage of embryo cells in this process. Additionally, raffinose family oligosaccharides (RFOs) are a type of functional oligosaccharides that is unique to plants. Some studies have shown that RFOs usually accumulate during the late stages of seed development and are closely related to seed vigor, dehydration tolerance, and storage tolerance (Leprince et al., 2017). In this study, soluble sugar contents increased gradually during the rapid dehydration stage, and 4 DEPs related to RFOs synthesis pathway involved in raffinose and stachyose biosynthesis, including UDP-glucose 4-epimerase (A0A1U8APY8, A0A1U8Q8U3), inositol 3-α-galactosyltransferase (A0A1U8B9X9), sucrose synthetase (A0A1U8APR5), and UDP-glucose-6-dehydrogenase (A0A1U8A842). Those enzymes synergistically promote the synthesis of RFOs. Therefore, some oligosaccharides may accumulate in lotus embryo to resist dehydration osmotic stress.

### Stress Response and Redox Homeostasis Status During Lotus Seed Dehydration Maturity

Cells usually suffer severe stress damage, lipid peroxidation, and macromolecules denaturation due to extreme dehydration during seed maturation and development. Biological macromolecules, including plasma membrane, enzymes, and nucleic acids, may be attacked by excessive ROS, which is the main factor causing seed aging and death. ROS is mainly eliminated by enzymatic or non-enzymatic antioxidants. In this study, stress physiological indicators indicated that H_2_O_2_ was the main ROS component accumulated in lotus embryos, and GSH and V_E_ act as the major antioxidants to resist oxidative stress during the seed dehydration process. Several antioxidant enzymes (SOD, APX, and GPX) were significantly downregulated during dehydration of lotus embryos. However, glutathione synthase (GS; A0A1U7ZUJ5) and two glutathione S-transferase (GST; A0A1U8A0H7, A0A1U7YZC7) and TRX (A0A1U7ZHC7) were significantly up-regulated in this process (Supplementary Figure 5). GS can promote glutathione production, and GST can catalyze GSH to chelate metal ions or combine with hydrophobic and electrophilic substrates to degrade the harmful substances of cells, helping cells resist oxidative damage (Zhang D. et al., 2015). TRX contains disulfide bonds with redox activity, which could regulate the redox state of cells through the reversible conversion of thiol-disulfide bonds. GST is positively related to the antioxidant capacity of plants and has been found in *Nicotiana tabacum*, rice, and *Arabidopsis* for its ability to resist oxidative stress (Gallé et al., 2009; Rezaei et al., 2013). It is speculated that GST may catalyze the chelation of GSH with metal ions to prevent metal ions from combining with ROS to turn into highly toxic hydroxyl radicals. The GSH pathway involving GS and GST actively responds to oxidative stress during the dehydration of lotus embryos. The above results further suggested that non-enzymatic antioxidants play a fundamental role in stress damage protection during water loss process in lotus embryos.

When undergoing desiccation, cells have to cope with significant changes in turgor pressure, which lead to cell shrinkage and mechanical damages (Scoffoni et al., 2014). ABA is found to be likely important in the acquisition of dehydration tolerance (DT) and drought tolerance during seed maturation and development (Umezawa et al., 2010; Hauser et al., 2011). ABA Insensitive (ABI) protein is a positive transcription factor in the ABA signaling pathway, which is involved in plant ABA signaling-mediated seed development or abiotic stress process (Gutierrez et al., 2007). In *Arabidopsis*, ABI5 can regulate dormancy and germination of seeds (Lopez-Molina et al., 2001; Maia et al., 2014). ABI5 is the hub of the seed survival gene regulatory network under drought conditions in *Medicago truncatula* (Verdier et al., 2013). The function of ABI5 in the process of seed maturity was subsequently revealed in *Pisum sativum*, suggesting that ABI5, which is related to the synthesis and accumulation of raffinose family RFOs and LEA protein, is an important regulator of maturity development and longevity in legume seeds (Zinsmeister et al., 2016). In *Physcomitrella patens*, knockouts of the *ABI3* gene that regulate ABA signaling were shown to affect DT acquisition of the seed (Khandelwal et al., 2010). Some *abi3-5* mutant seeds failed to acquire DT and displayed a reduced longevity (Sugliani et al., 2009), while two *Mtabi3* mutants in *Medicago* led to a lower expression of LEA proteins, abnormal chlorophyll degradation in cotyledons, and defective in chromatin compaction and nuclear size reduction (Delahaie et al., 2013; Delmas et al., 2013; van Zanten et al., 2014). In lotus seed embryos, ABA contents were increased at 15 and 30 DAP, and three ABI5 proteins (A0A1U8B646, A0A1U7ZTI7, A0A1U7YYQ4) were significantly differently expressed at the late dehydration stage, indicating that ABA signaling can induce embryo dehydration and regulate downstream biological events in lotus. During seed drying, the accumulation of macromolecules such as oligosaccharides, LEA, or HSPs proteins greatly increases cytoplasmic viscosity and usually causes the formation of bioglasses. Thus, bioglasses have been suggested to provide intracellular protection against the denaturation of large molecules to stabilize plasma membranes. The major seed proteins involved in bioglasses formation should be LEA proteins (Shih et al., 2008). The LEA proteins are a family of protective proteins that accumulate at the late stages of seed development. On the one hand, it can take advantage of its highly hydrophilic characteristics to prevent excessive loss of water from the cell, thereby stabilizing the membrane structure and renaturing unfolded proteins. On the other hand, it can reduce oxidative stress damage to cells by chelating metal ions (Tunnacliffe and Wise, 2007; Yang et al., 2019). Some studies have been proved that the LEA family could maintain the vitality of seed embryos and improve cell resistance during storage in maize, alfalfa, *Arabidopsis*, and wheat (Kalemba and Pukacka, 2008; Rajjou et al., 2008; Wu et al., 2011; Chatelain et al., 2012). *ATEM1* and *ATEM6* (LEA family) knockout mutations of *Arabidopsis* display premature seed dehydration and maturation (Manfre et al., 2006), and recent studies have demonstrated that seed longevity is reduced when the seed-specific dehydrins were decreased in *A. thaliana* (Rajjou et al., 2008; Hundertmark et al., 2011). In rice, LEA proteins and their mRNAs accumulate to high concentrations during seed development and maturation (Sano et al., 2013), and glutathione-related proteins, DNA-damage-repair proteins and LEA protein might be correlated with seed storability (Gao et al., 2016). Furthermore, HSPs, as molecular chaperones, are mainly involved in the processing and transportation of nascent peptides, and also, in the repair and degradation of damaged proteins under stress. HSP accumulated at the late stage of maturation in many seed embryos, such as *Arabidopsis*, sunflower, and pea (Coca et al., 1994; DeRocher and Vierling, 1994; Wehmeyer et al., 1996), and had a close relationship with plant seed resistance and storage tolerance (Downs et al., 1999; Guo et al., 2007). In this study, 3 ABI5-like, 8 LEA proteins (significantly up-regulated), and 17 HSPs (8 up-regulated and 9 down-regulated) showed obvious expression changes during dehydration process in lotus embryos, in particular, RAB18 (response to ABA) dehydrin was the most abundant protein among all DEPs. These findings suggested that ABA signaling and the accumulation of LEA and HSPs proteins may be the key factors to ensure the continuous dehydration and storage tolerance of lotus seed embryos.

### DEPs Involved in Protein Modification and Degradation

Histone acetylation modification is a key mechanism of plant gene transcriptional regulation and is closely related to gene expression. Histone deacetylase (HDAC) could participate in the remodeling of chromatin structure, playing an important role in the epigenetic regulation of plant genes (Zhang and Zhong, 2014). Numerous studies have reported that HDAC could regulate the maturation and development of seeds or abiotic stress in plants (Wu et al., 2000; Zhou et al., 2004; Gao et al., 2015). The *AtHD2C*-transgenic *Arabidopsis* are less sensitive to ABA, high salt, and drought stresses (To et al., 2011). In rice, overexpression of *SRT1* gene could enhance the ability of rice to resist oxidative stress (Zhong et al., 2013). In our study, we identified three types of HDAC family members (A0A1U7YW34, A0A1U8Q4V5, A0A1U8BLQ1), and their expressions were significantly up-regulated during maturation drying, indicating that HDAC positively regulating the maturation and development of lotus embryos. The up-regulated HDAC may respond to the ABA signaling pathway and enhance the tolerance of lotus embryo cells during continuous dehydration through regulation of gene transcription. Plants usually remove some damaged or useless organelles and proteins through autophagy (on type of programed cell death, PCD) to maintain the normal physiological activities when growing under abiotic stress conditions. However, excessive cellular autophagy may lead to serious protein degradation and cell death, causing severe damage to cells (Iakimova et al., 2005). Cysteine proteinase (CP) and subtilisin-like protease (SBT), as important proteolytic enzymes related to plant PCD events, are involved in the removal of damaged cells and the hydrolysis of most storage proteins in plants (Hatsugai et al., 2009; Tran et al., 2014). Cysteine proteinase inhibitor (CPI) can prevent the PCD events, such as hydrolysis of storage proteins or excessive autophagy, by inhibiting CP activity (Massonneau et al., 2005). In *Zea mays*, CPI was accumulated in mature seed embryos, which regulates the turnover of storage proteins during seed development (Wu et al., 2011). In our study, remarkably, CPI was significantly up-regulated, while CP and SBT were downregulated in lotus embryos. The expression changes of the above proteins suggested that PCD events are involved in lotus embryo morphogenesis, but PCD and storage proteolysis events are weakened during seed dehydration and maturity process, which beneficial to the nutrient accumulation and storage in embryos.

### DEPs Involved in DNA Repair

DNA damage, such as nucleobase modification and DNA strand breaking, usually occurs when seeds suffered from dehydration or some adverse environmental conditions exacerbating seed deterioration and aging (Cheah and Osborne, 1978). DNA repair enzymes could effectively repair damaged DNA and maintain normal physiological activities of cells. A previous study found that DNA repair proteins are downregulated in rice embryos which are not resistant to storage, indicating that these proteins have a certain relationship with the storage tolerance of seed embryos (Manfre et al., 2006). High mobility group proteins (HMG) are widely involved in gene expression and regulation, including DNA replication, transcription, recombination, and DNA repair process (Kalyna et al., 2003; Grasser et al., 2007). In addition, the accumulation of histones was also found in *Brachypodium distachyon* embryos during seed maturation (Wolny et al., 2017). These proteins play an important role in DNA repair by rearranging chromatin structure to reduce ROS damage during seed maturation drying (Kalemba and Pukacka, 2008). In this study, we found that a number of proteins related to DNA repair were significantly up-regulated, including DNA damage repair proteins (A0A1U7ZYD0), HMG proteins (A0A1U7ZKY2, A0A1U8ATJ2, A0A1U7Z4F5), and histones (A0A1U7Z5K0, A0A1U8AWH0), indicating that DNA regulation and repair mechanism play a positive role in maintaining normal physiological activities and cell defense during lotus embryos dehydration and maturity process.

## Conclusions

In this study, a TMT-based quantitative proteomic approach was used to reveal differential protein profiling during the lotus embryo continuous dehydration and maturity process. Bioinformatics analysis showed that environmental stimuli, stress responses, and protein processing related biological processes are vigorous, and carbohydrate metabolism, protein processing in ER, DNA repair, and antioxidative events have a positive response to lotus embryo dehydration. Furthermore, H_2_O_2_ was the main ROS component inducing oxidative stress damage, and GSH and V_E_ acted as the major antioxidant to maintain the REDOX balance of lotus embryo. ABA signal and the accumulation of oligosaccharides, LEA, and HSPs may be the key factors to ensure the continuous dehydration and storage tolerance of lotus seed embryo. Future studies focusing on characterizing the biological significance of LEA, HSPs, and oligosaccharide synthesis-related key proteins will be highly valuable in designing molecular breeding or engineering programs for enhancing plant tolerance to dehydration.

## Data Availability Statement

The original contributions presented in the study are publicly available. This data can be found here: ProteomeXchange via the PRIDE database, with the accession number PXD029187.

## Author Contributions

DZ conceived the original research plans and designed the experiments. TL, JS, and SL collected and analyzed the data. DZ and LR wrote the manuscript. All authors have read and approved the final manuscript.

## Funding

This work was sponsored by the National Natural Science Foundation of China (Grant Nos. 31971705 and 31870686) and Natural Science Foundation of Shanghai (Grant No. 21ZR1434200).

## Conflict of Interest

The authors declare that the research was conducted in the absence of any commercial or financial relationships that could be construed as a potential conflict of interest.

## Publisher's Note

All claims expressed in this article are solely those of the authors and do not necessarily represent those of their affiliated organizations, or those of the publisher, the editors and the reviewers. Any product that may be evaluated in this article, or claim that may be made by its manufacturer, is not guaranteed or endorsed by the publisher.

## Supplementary Material

Supplementary Figure 1 Gene Oncology (GO) terms annotation of all differentially expressed proteins (DEPs) in lotus embryos during seed dehydration maturity. **(A)** GO functional annotation of up-regulated DEPs; **(B)** GO functional annotation of down-regulated DEPs.

Supplementary Figure 2 AgriGO significant enrichment functional annotation of DEPs from lotus embryo during seed dehydration maturity.

Supplementary Figure 3 KEGG pathway term significant enrichment analysis of all DEPs in lotus embryos during seed dehydration maturity.

Supplementary Figure 4 KEGG pathway significant enrichment analysis of DEPs from lotus embryo during seed dehydration and maturity. **(A)** Significant enrichment analysis of up-regulated DEPs; **(B)** significant enrichment analysis of down-regulated DEPs.

Supplementary Figure 5 Parallel reaction monitoring quantitative verification of eighteen proteins from tandem mass tags (TMT) proteomics data. Asterisks indicate statistically significant differences (^***^*p* < 0.001; ^**^*p* < 0.01; ^*^*p* < 0.05) between lotus embryos at different dehydration stages.

Supplementary Figure 6 Carbohydrates, energy, and antioxidation system regulation model of key DEPs from lotus embryo during seed dehydration maturity.

Supplementary Table 1 Detailed quantitative information of identified proteins from lotus embryo during seed dehydration maturity.

Supplementary Table 2 Detailed quantitative information of screened differentially expressed proteins from lotus embryo during seed dehydration maturity. The orange background represents up-regulated DEPs and the green background represents downregulated DEPs.

Supplementary Table 3 Functional classification of key DEPs from lotus embryo during dehydration maturity.

Supplementary Table 4 Correlation analysis of stress physiological indices of lotus embryo during dehydration maturity.

## References

[B1] Bo C. Geng X. Zhang J. Sai L. Zhang Y. Yu G.. (2020). Comparative proteomic analysis of silica-induced pulmonary fibrosis in rats based on tandem mass tag (TMT) quantitation technology. PLoS ONE 15:e0241310. 10.1371/journal.pone.0241310 33119648 PMC7595299

[B2] Burke M. (1986). The Glassy State and Survival of Anhydrous Biological Systems. Ithaca, NY: Cornell University Press.

[B3] Chatelain E. Hundertmark M. Leprince O. Gall S. L. Satour P. Deligny-penninck S.. (2012). Temporal profiling of the heat-stable proteome during late maturation of *Medicago truncatula* seeds identifies a restricted subset of late embryogenesis abundant proteins associated with longevity. Plant Cell Environ. 35, 1440–1455. 10.1111/j.1365-3040.2012.02501.x 22380487

[B4] Cheah K. S. E. Osborne D. J. (1978). DNA lesions occur with loss of viability in embryos of ageing rye seed. Nature 272, 593–599. 10.1038/272593a0 19213149

[B5] Coca M. A. Almoguera C. Jordano J. (1994). Expression of sunflower low-molecular-weight heat-shock proteins during embryogenesis and persistence after germination: localization and possible functional implications. Plant Mol. Biol. 25, 479–492. 10.1007/BF00043876 8049372

[B6] Debeaujon I. Léon-Kloosterziel K. M. Koornneef M. (2000). Influence of the testa on seed dormancy, germination, and longevity in *Arabidopsis*. Plant Physiol. 122, 403–414. 10.1104/pp.122.2.403 10677433 PMC58877

[B7] Dekkers B. J. W. Costa M. C. D. Maia J. Bentsink L. Ligterink W. Hilhorst H. W. M. (2015). Acquisition and loss of desiccation tolerance in seeds: From experimental model to biological relevance. Planta 241, 563–577. 10.1007/s00425-014-2240-x 25567203

[B8] Delahaie J. Hundertmark M. Bove J. Leprince O. Rogniaux H. Buitink J. (2013). LEA polypeptide profiling of recalcitrant and orthodox legume seeds reveals ABI3-regulated LEA protein abundance linked to desiccation tolerance. J. Exp. Bot. 64, 4559–4573. 10.1093/jxb/ert274 24043848 PMC3808335

[B9] Delmas F. Sankaranarayanan S. Deb S. Widdup E. Bournonville C. Bollier N.. (2013). ABI3 controls embryo degreening through Mendel's locus. Proc. Natl. Acad. Sci. U.S.A. 110:E3888. 10.1073/pnas.1308114110 24043799 PMC3791760

[B10] Delseny M. Bies-Etheve N. Carles C. Hull G. Vicient C. Raynal M.. (2001). Late Embryogenesis Abundant (LEA) protein gene regulation during *Arabidopsis* seed maturation. J. Plant Physiol. 158, 419–427. 10.1078/0176-1617-00353

[B11] DeRocher A. E. Vierling E. (1994). Developmental control of small heat shock protein expression during pea seed maturation. Plant J. 5, 93–102. 10.1046/j.1365-313X.1994.5010093.x

[B12] Downs C. A. Coleman J. S. Heckathorn S. A. (1999). The chloroplast 22-Ku heat-shock protein: a lumenal protein that associates with the oxygen evolving complex and protects photosystem II during heat stress. J. Plant Physiol. 155, 477–487. 10.1016/S0176-1617(99)80042-X

[B13] Gallé Á. Csiszár J. Secenji M. Guóth A. Cseuz L. Tari I.. (2009). Glutathione transferase activity and expression patterns during grain filling in flag leaves of wheat genotypes differing in drought tolerance: response to water deficit. J. Plant Physiol. 166, 1878–1891. 10.1016/j.jplph.2009.05.016 19615785

[B14] Gao J. Fu H. Zhou X. Chen Z. Luo Y. Cui B.. (2016). Comparative proteomic analysis of seed embryo proteins associated with seed storability in rice (*Oryza sativa* L.) during natural aging. Plant Physiol. Bioch. 103, 31–44. 10.1016/j.plaphy.2016.02.026 26950923

[B15] Gao M. Li X. Huang J. Gropp G. M. Gjetvaj B. Lindsay D. L.. (2015). SCARECROW-LIKE15 interacts with HISTONE DEACETYLASE19 and is essential for repressing the seed maturation programme. Nat. Commun. 6:7243. 10.1038/ncomms8243 26129778 PMC4507008

[B16] Gong M. Zhang H. Wu D. Zhang Z. Zhang J. Bao D.. (2021). Key metabolism pathways and regulatory mechanisms of high polysaccharide yielding in *Hericium erinaceus*. BMC Genomics 22:160. 10.1186/s12864-021-07480-x 33676419 PMC7937317

[B17] Grasser K. D. Launholt D. Grasser M. (2007). High mobility group proteins of the plant HMGB family: dynamic chromatin modulators. Genet. Resour. Crop Ev. 1769, 346–357. 10.1016/j.bbaexp.2006.12.004 17316841

[B18] Guo H. (2009). Cultivation of lotus (*Nelumbo nucifera* Gaertn. ssp. *nucifera*) and its utilization in China. Genet. Resour. Crop Ev. 56, 323–330. 10.1007/s10722-008-9366-2

[B19] Guo S. Zhou H. Zhang X. Li X. Meng Q. (2007). Overexpression of *CaHSP26* in transgenic tobacco alleviates photoinhibition of PSII and PSI during chilling stress under low irradiance. J. Plant Physiol. 164, 126–136. 10.1016/j.jplph.2006.01.004 16513207

[B20] Gutierrez L. van Wuytswinkel O. Castelain M. Bellini C. (2007). Combined networks regulating seed maturation. Trends Plant Sci. 12, 294–300. 10.1016/j.tplants.2007.06.003 17588801

[B21] Hatsugai N. Iwasaki S. Tamura K. Kondo M. Kentaro F. Kimi O.. (2009). A novel membrane fusion-mediated plant immunity against bacterial pathogens. Gene. Dev. 23, 2496–2506. 10.1101/gad.1825209 19833761 PMC2779742

[B22] Hauser F. Waadt R. Schroeder J. I. (2011). Evolution of abscisic acid synthesis and signaling mechanisms. Curr. Biol. 21, R346–R355. 10.1016/j.cub.2011.03.015 21549957 PMC3119208

[B23] Hoekstra F. A. Golovina E. A. Buitink J. (2001). Mechanisms of plant desiccation tolerance. Trends Plant Sci. 6, 431–438. 10.1016/S1360-1385(01)02052-0 11544133

[B24] Huang S. Tang X. Zhang L. Rui F. (2003). Thermotolerance and activity of antioxidative enzymes in lotus seeds. J. Plant Physiol. Mol. Biol. 5, 421–424. (in Chinese).

[B25] Hundertmark M. Buitink J. Leprince O. Hincha D. K. (2011). The reduction of seed-specific dehydrins reduces seed longevity in *Arabidopsis thaliana*. Seed Sci. Res. 21, 165–173. 10.1017/S096025851100007930886898

[B26] Iakimova E. Kapchina-Toteva V. de Jong A. Atanassov A. Woltering E. (2005). Involvement of ethylene, oxidative stress and lipid-derived signals in cadmium-induced programmed cell death in tomato suspension cells. BMC Plant Biol. 5:S19. 10.1186/1471-2229-5-S1-S19 17079154

[B27] Kalemba E. M. Pukacka S. (2008). Changes in late embryogenesis abundant proteins and a small heat shock protein during storage of beech (*Fagus sylvatica* L.) seeds. Environ. Exp. Bot. 63, 274–280. 10.1016/j.envexpbot.2007.12.011

[B28] Kalyna M. Lopato S. Barta A. (2003). Ectopic expression of atRSZ33 reveals its function in splicing and causes pleiotropic changes in development. Mol. Biol. Cell 14, 3565–3577. 10.1091/mbc.e03-02-0109 12972547 PMC196550

[B29] Khandelwal A. Cho S. H. Marella H. Sakata Y. Perroud P. F. Pan A.. (2010). Role of ABA and ABI3 in desiccation tolerance. Science 327:546. 10.1126/science.1183672 20110497

[B30] Kranner I. Birtić S. Anderson K. M. Pritchard H. W. (2006). Glutathione half-cell reduction potential: a universal stress marker and modulator of programmed cell death? Free Radical Bio. Med. 40, 2155–2165. 10.1016/j.freeradbiomed.2006.02.013 16785029

[B31] Kruger N. J. von Schaewen A. (2003). The oxidative pentose phosphate pathway: structure and organisation. Curr. Opin. Plant Biol. 6, 236–246. 10.1016/S1369-5266(03)00039-6 12753973

[B32] Lee J. Koh H. (2011). A label-free quantitative shotgun proteomics analysis of rice grain development. Proteome Sci. 9:61. 10.1186/1477-5956-9-61 21957990 PMC3190340

[B33] Leprince O. Pellizzaro A. Berriri S. Buitink J. (2017). Late seed maturation: drying without dying. J. Exp. Bot. 68, 827–841. 10.1093/jxb/erw363 28391329

[B34] Lopez-Molina L. Mongrand S. Chua N. (2001). A postgermination developmental arrest checkpoint is mediated by abscisic acid and requires the ABI5 transcription factor in *Arabidopsis*. Proc. Natl. Acad. Sci. U.S.A. 98:4782. 10.1073/pnas.081594298 11287670 PMC31911

[B35] Maia J. Dekkers B. J. W. Dolle M. J. Ligterink W. Hilhorst H. W. M. (2014). Abscisic acid (ABA) sensitivity regulates desiccation tolerance in germinated *Arabidopsis* seeds. New Phytol. 203, 81–93. 10.1111/nph.12785 24697728

[B36] Manfre A. J. Lanni L. M. Marcotte W. R. (2006). The *Arabidopsis* group 1 late embryogenesis abundant protein ATEM6 is required for normal seed development. Plant Physiol. 140, 140–149. 10.1104/pp.105.072967 16361514 PMC1326038

[B37] Massonneau A. Condamine P. Wisniewski J. Zivy M. Rogowsky P. M. (2005). Maize cystatins respond to developmental cues, cold stress and drought. BBA Gene Struct. Expr. 1729, 186–199. 10.1016/j.bbaexp.2005.05.004 15979170

[B38] Ming R. VanBuren R. Liu Y. Yang M. Han Y. Li L.. (2013). Genome of the long-living sacred lotus (*Nelumbo nucifera* Gaertn.). Genome Biol. 14:R41. 10.1186/gb-2013-14-5-r41 23663246 PMC4053705

[B39] Moro C. F. Fukao Y. Shibato J. Rakwal R. Agrawal G. K. Shioda S.. (2015). Immature seed endosperm and embryo proteomics of the Lotus (*Nelumbo Nucifera* Gaertn.) by one-dimensional gel-based tandem mass spectrometry and a comparison with the mature endosperm proteome. Proteomes 3, 184–235. 10.3390/proteomes3030184 28248268 PMC5217381

[B40] Mukherjee D. Khatua T. N. Venkatesh P. Saha B. P. Mukherjee P. K. (2010). Immunomodulatory potential of rhizome and seed extracts of *Nelumbo nucifera* Gaertn. J. Ethnopharmacol. 128, 490–494. 10.1016/j.jep.2010.01.015 20079418

[B41] Prieto-Dapena P. Castaño R. Almoguera C. Jordano J. (2006). Improved resistance to controlled deterioration in transgenic seeds. Plant Physiol. 142, 1102–1112. 10.1104/pp.106.087817 16998084 PMC1630740

[B42] Rajjou L. Lovigny Y. Groot S. P. C. Belghazi M. Job C. Job D. (2008). Proteome-Wide characterization of seed aging in *Arabidopsis*: a comparison between artificial and natural aging protocols. Plant Physiol. 148, 620–641. 10.1104/pp.108.123141 18599647 PMC2528126

[B43] Rezaei M. K. Shobbar Z. Shahbazi M. Abedini R. Zare S. (2013). Glutathione S-transferase (GST) family in barley: Identification of members, enzyme activity, and gene expression pattern. J. Plant Physiol. 170, 1277–1284. 10.1016/j.jplph.2013.04.005 23664583

[B44] Sano N. Masaki S. Tanabata T. Yamada T. Hirasawa T. Kanekatsu M. (2013). Proteomic analysis of stress-related proteins in rice seeds during the desiccation phase of grain filling. Plant Biotechnol. Nar. 30, 147–156. 10.5511/plantbiotechnology.13.0207a

[B45] Sattler S. E. Gilliland L. U. Magallanes-Lundback M. Pollard M. DellaPenna D. (2004). Vitamin E is essential for seed longevity and for preventing lipid peroxidation during germination. Plant Cell 16, 1419–1432. 10.1105/tpc.021360 15155886 PMC490036

[B46] Scharte J. Schön H. Tjaden Z. Weis E. von Schaewen A. (2009). Isoenzyme replacement of glucose-6-phosphate dehydrogenase in the cytosol improves stress tolerance in plants. Proc. Natl. Acad. Sci. U.S.A. 106:8061. 10.1073/pnas.0812902106 19416911 PMC2683143

[B47] Scheibe R. (2004). Malate valves to balance cellular energy supply. Physiol. Plant. 120, 21–26. 10.1111/j.0031-9317.2004.0222.x 15032873

[B48] Scoffoni C. Vuong C. Diep S. Cochard H. Sack L. (2014). Leaf shrinkage with dehydration: coordination with hydraulic vulnerability and drought tolerance. Plant Physiol. 164, 1772–1788. 10.1104/pp.113.221424 24306532 PMC3982740

[B49] Shen-Miller J. (2002). Sacred lotus, the long-living fruits of *China Antique*. Seed Sci. Res. 12, 131–143. 10.1079/SSR200211224363819

[B50] Shen-Miller J. Lindner P. Xie Y. Villa S. Wooding K. Clarke S. G.. (2013). Thermal-stable proteins of fruit of long-living sacred lotus *Nelumbo nucifera* Gaertn var. China antique. Trop. Plant Biol. 6, 69–84. 10.1007/s12042-013-9124-2 24363819 PMC3869599

[B51] Shih M. Hoekstra F. A. Hsing Y. C. (2008). Late Embryogenesis Abundant Proteins. Pittsburgh, PA: Academic Press. 10.1016/S0065-2296(08)00404-7

[B52] Sugliani M. Rajjou L. Clerkx E. J. M. Koornneef M. Soppe W. J. J. (2009). Natural modifiers of seed longevity in the *Arabidopsis* mutants *abscisic acid insensitive3-5* (*abi3-5*) and *leafy cotyledon1-3* (*lec1-3*). New Phytol. 184, 898–908. 10.1111/j.1469-8137.2009.03023.x 19754639

[B53] To T. K. Kim J. Matsui A. Kurihara Y. Morosawa T. Ishida J.. (2011). *Arabidopsis* HDA6 regulates locus-directed heterochromatin silencing in cooperation with MET1. PLoS Genet. 7:e1002055. 10.1371/journal.pgen.1002055 21552333 PMC3084210

[B54] Tran V. Weier D. Radchuk R. Thiel J. Radchuk V. (2014). Caspase-like activities accompany programmed cell death events in developing barley grains. PLoS ONE 9:e109426. 10.1371/journal.pone.0109426 25286287 PMC4186829

[B55] Tunnacliffe A. Wise M. J. (2007). The continuing conundrum of the LEA proteins. Naturwissenschaften 94, 791–812. 10.1007/s00114-007-0254-y 17479232

[B56] Umezawa T. Nakashima K. Miyakawa T. Kuromori T. Tanokura M. Shinozaki K.. (2010). Molecular basis of the core regulatory network in ABA responses: sensing, signaling and transport. Plant Cell Physiol. 51, 1821–1839. 10.1093/pcp/pcq156 20980270 PMC2978318

[B57] van Zanten M. Zöll C. Wang Z. Philipp C. Carles A. Li Y.. (2014). HISTONE DEACETYLASE 9 represses seedling traits in *Arabidopsis thaliana* dry seeds. Plant J. 80, 475–488. 10.1111/tpj.12646 25146719

[B58] Verdier J. Lalanne D. Pelletier S. Torres-Jerez I. Righetti K. Bandyopadhyay K.. (2013). A regulatory network-based approach dissects late maturation processes related to the acquisition of desiccation tolerance and longevity of *Medicago truncatula* seeds. Plant Physiol. 163, 757–774. 10.1104/pp.113.222380 23929721 PMC3793056

[B59] Wang L. Fu J. Li M. Fragner L. Weckwerth W. Yang P. (2016). Metabolomic and proteomic profiles reveal the dynamics of primary metabolism during seed development of Lotus (*Nelumbo nucifera*). Front. Plant Sci. 7:750. 10.3389/fpls.2016.00750 27375629 PMC4894879

[B60] Wang Y. Fan G. Liu Y. Sun F. Shi C. Liu X.. (2013). The sacred lotus genome provides insights into the evolution of flowering plants. Plant J. 76, 557–567. 10.1111/tpj.12313 23952714

[B61] Wehmeyer N. Hernandez L. D. Finkelstein R. R. Vierling E. (1996). Synthesis of small heat-shock proteins is part of the developmental program of late seed maturation. Plant Physiol. 112, 747–757. 10.1104/pp.112.2.747 8883386 PMC157999

[B62] Wiśniewski J. R. Zougman A. Nagaraj N. Mann M. (2009). Universal sample preparation method for proteome analysis. Nat. Methods 6, 359–362. 10.1038/nmeth.1322 19377485

[B63] Wolny E. Braszewska-Zalewska A. Kroczek D. Hasterok R. (2017). Histone H3 and H4 acetylation patterns are more dynamic than those of DNA methylation in *Brachypodium distachyon* embryos during seed maturation and germination. Protoplasma 254, 2045–2052. 10.1007/s00709-017-1088-x 28236006 PMC5610208

[B64] Wu K. Tian L. Malik K. Brown D. Miki B. (2000). Functional analysis of HD2 histone deacetylase homologues in *Arabidopsis thaliana*. Plant J. 22, 19–27. 10.1046/j.1365-313x.2000.00711.x 10792817

[B65] Wu X. Liu H. Wang W. Chen S. Hu X. Li C. (2011). Proteomic analysis of seed viability in maize. Acta Physiol. Plant. 33, 181–191. 10.1007/s11738-010-0536-426630375

[B66] Yang Z. Sheng J. Lv K. Ren L. Zhang D. (2019). Y_2_SK_2_ and SK_3_ type dehydrins from *Agapanthus praecox* can improve plant stress tolerance and act as multifunctional protectants. Plant Sci. 284, 143–160. 10.1016/j.plantsci.2019.03.012 31084867

[B67] Zhang D. Ren L. Chen G. Zhang J. Reed B. M. Shen X. (2015). ROS-induced oxidative stress and apoptosis-like event directly affect the cell viability of cryopreserved embryogenic callus in *Agapanthus praecox*. Plant Cell Rep. 34, 1499–1513. 10.1007/s00299-015-1802-0 26104871

[B68] Zhang D. Ren L. Yue J. Wang L. Zhuo L. Shen X. (2013). A comprehensive analysis of flowering transition in *Agapanthus praecox* ssp. orientalis Leighton by using transcriptomic and proteomic techniques. J. Proteomics 80, 1–25. 10.1016/j.jprot.2012.12.028 23333928

[B69] Zhang J. Zhong Q. (2014). Histone deacetylase inhibitors and cell death. Cell. Mol. Life Sci. 71, 3885–3901. 10.1007/s00018-014-1656-6 24898083 PMC4414051

[B70] Zhang N. Chen F. Huo W. Cui D. (2015). Proteomic analysis of middle and late stages of bread wheat (*Triticum aestivum* L.) grain development. Front. Plant Sci. 6:735. 10.3389/fpls.2015.00735 26442048 PMC4569854

[B71] Zhang S. Ghatak A. Bazargani M. M. Bajaj P. Varshney R. K. Chaturvedi P.. (2021). Spatial distribution of proteins and metabolites in developing wheat grain and their differential regulatory response during the grain filling process. Plant J. 107, 669–687. 10.1111/tpj.15410 34227164 PMC9291999

[B72] Zhong X. Zhang H. Zhao Y. Sun Q. Hu Y. Peng H.. (2013). The rice NAD^+^-dependent histone deacetylase OsSRT1 targets preferentially to stress- and metabolism-related genes and transposable elements. PLoS ONE 8:e66807. 10.1371/journal.pone.0066807 23825566 PMC3692531

[B73] Zhou C. Labbe H. Sridha S. Wang L. Tian L. Latoszek-Green M.. (2004). Expression and function of HD2-type histone deacetylases in *Arabidopsis* development. Plant J. 38, 715–724. 10.1111/j.1365-313X.2004.02083.x 15144374

[B74] Zinsmeister J. Lalanne D. Terrasson E. Chatelain E. Vandecasteele C. Vu B. L.. (2016). ABI5 is a regulator of seed maturation and longevity in legumes. Plant Cell 28, 2735–2754. 10.1105/tpc.16.00470 27956585 PMC5155344

