# Supplementary figures and images for: TMT-Based Quantitative Proteomic Analysis Reveals the Physiological Regulatory Networks of Embryo Dehydration Protection in Lotus (Nelumbo nucifera)
Source: Front Plant Sci. 2021 Dec 17;12:792057. doi: 10.3389/fpls.2021.792057 (PMC8718645; doi:10.3389/fpls.2021.792057)

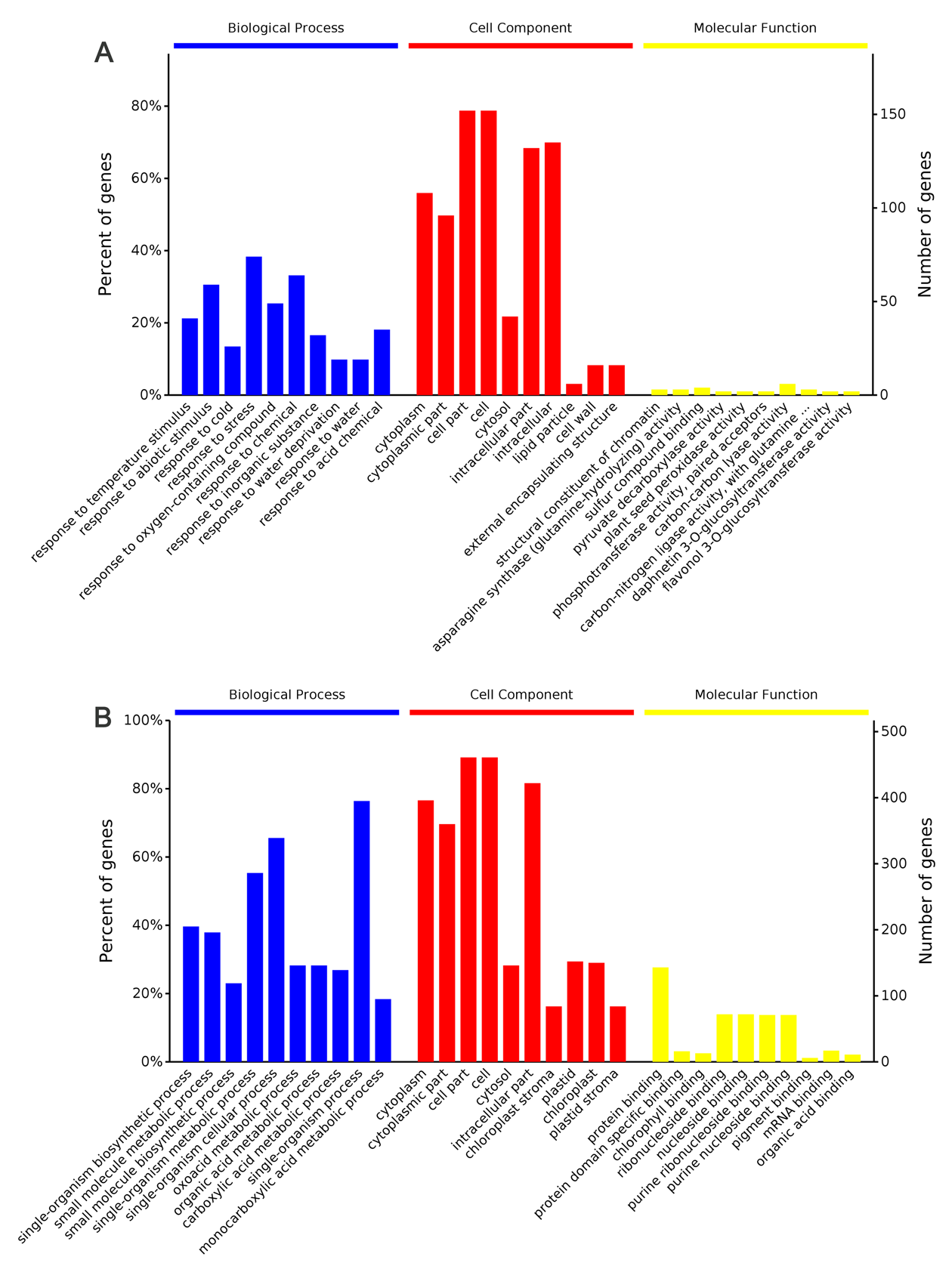

Supplement: Supplementary Figure 1 — Gene Oncology (GO) terms annotation of all differentially expressed proteins (DEPs) in lotus embryos during seed dehydration maturity. (A) GO functional annotation of up-regulated DEPs; (B) GO functional annotation of down-regulated DEPs. [file Image_1.TIF]

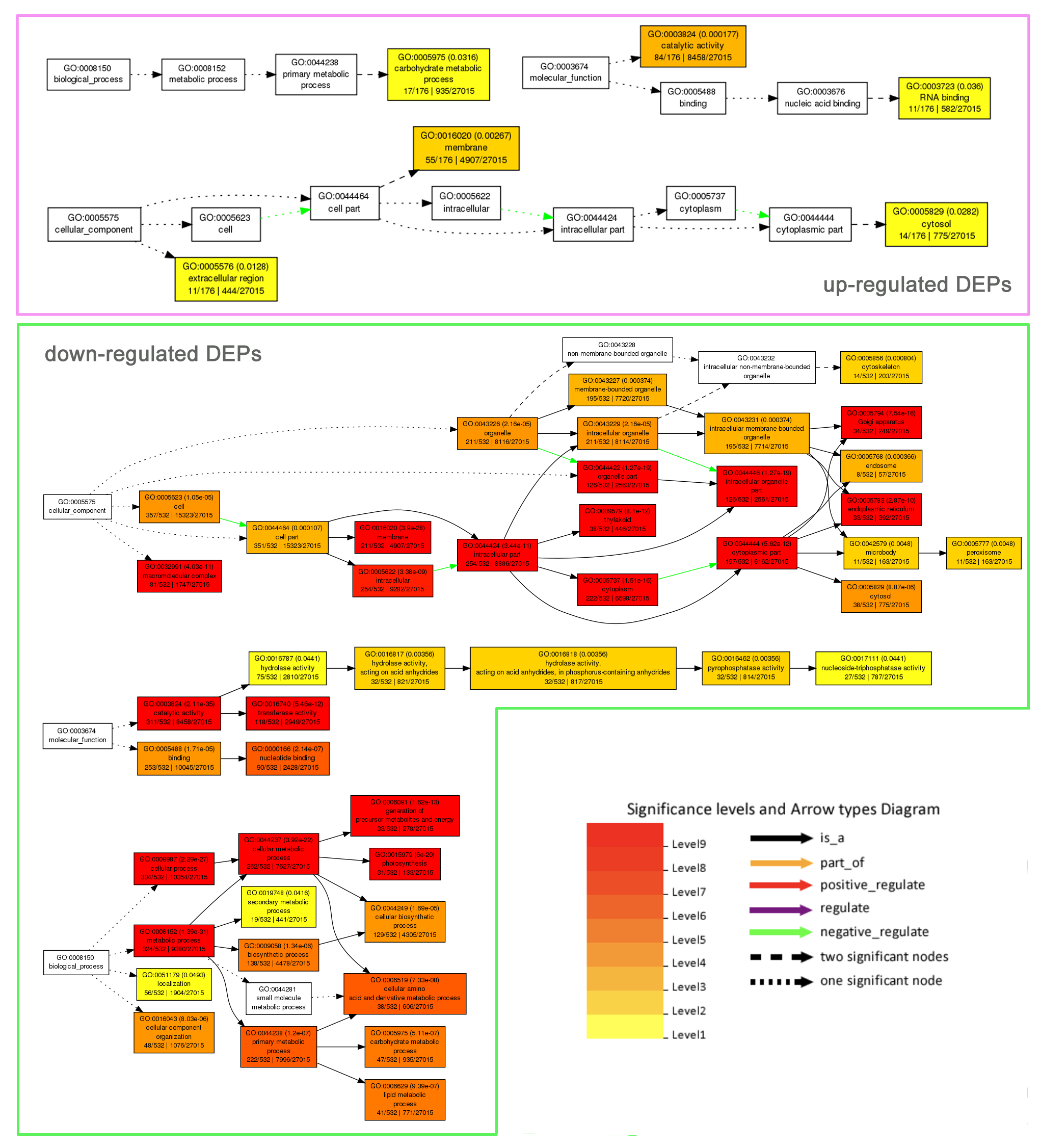

Supplement: Supplementary Figure 2 — AgriGO significant enrichment functional annotation of DEPs from lotus embryo during seed dehydration maturity. [file Image_2.TIF]

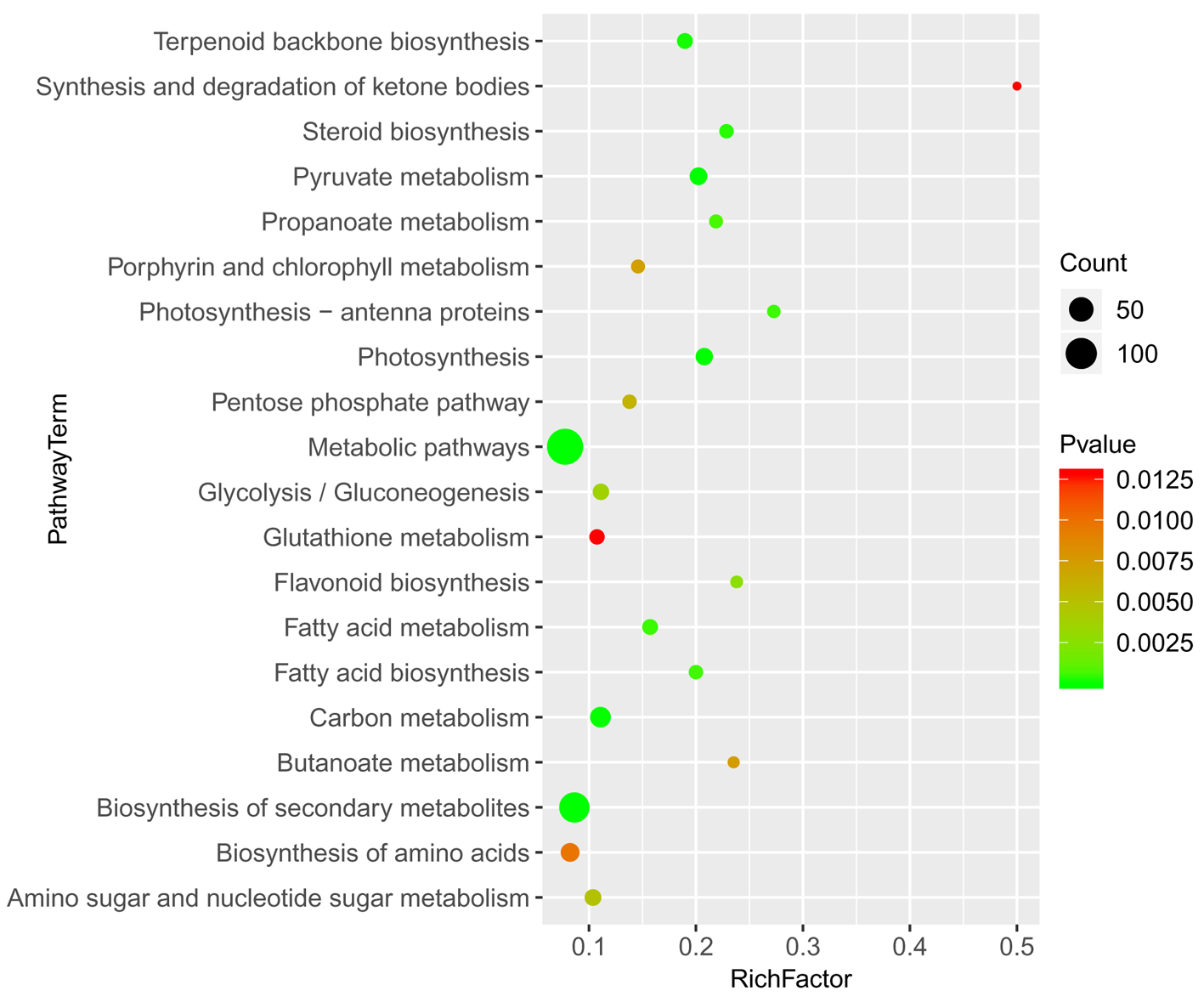

Supplement: Supplementary Figure 3 — KEGG pathway term significant enrichment analysis of all DEPs in lotus embryos during seed dehydration maturity. [file Image_3.TIF]

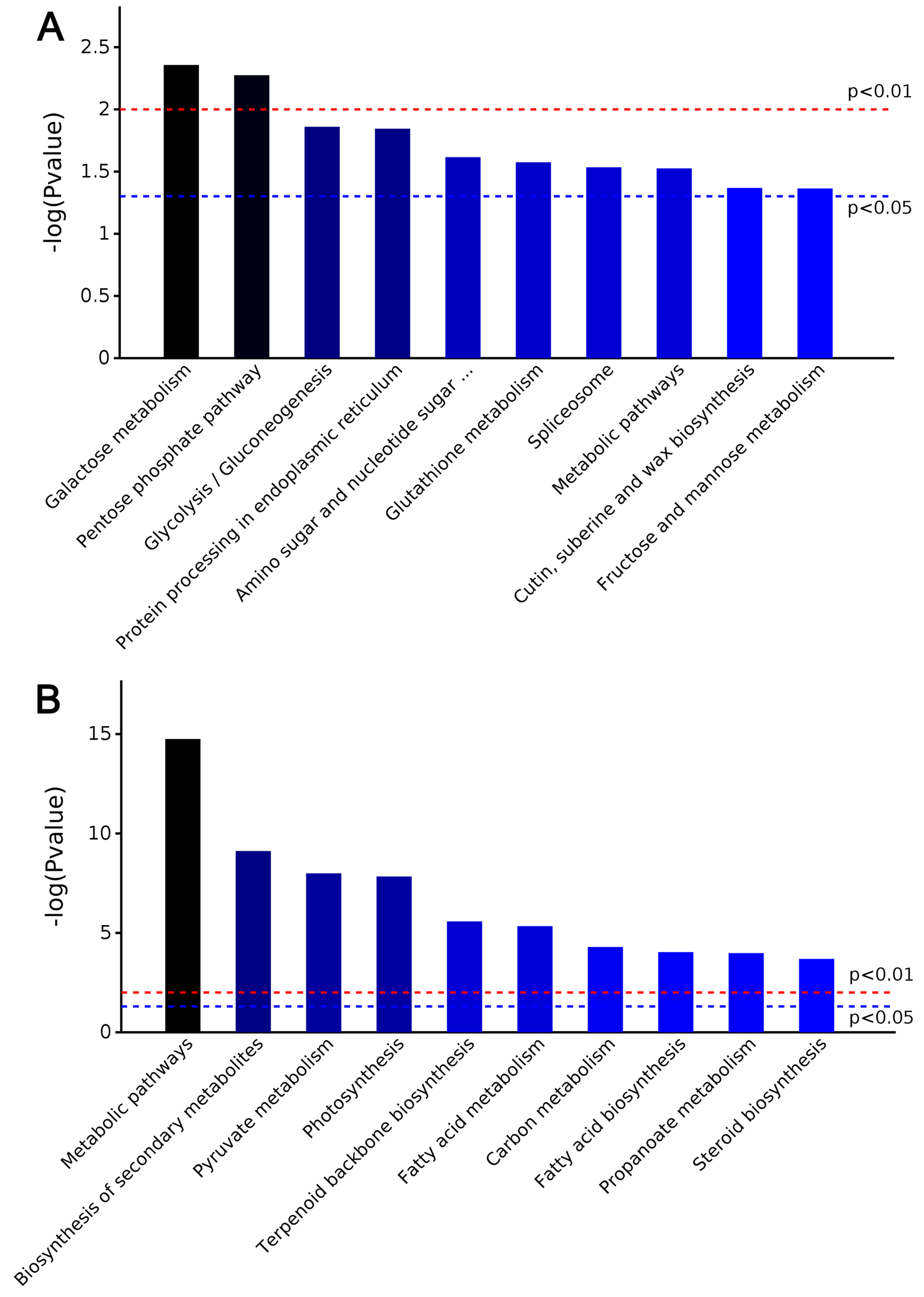

Supplement: Supplementary Figure 4 — KEGG pathway significant enrichment analysis of DEPs from lotus embryo during seed dehydration and maturity. (A) Significant enrichment analysis of up-regulated DEPs; (B) significant enrichment analysis of down-regulated DEPs. [file Image_4.TIF]

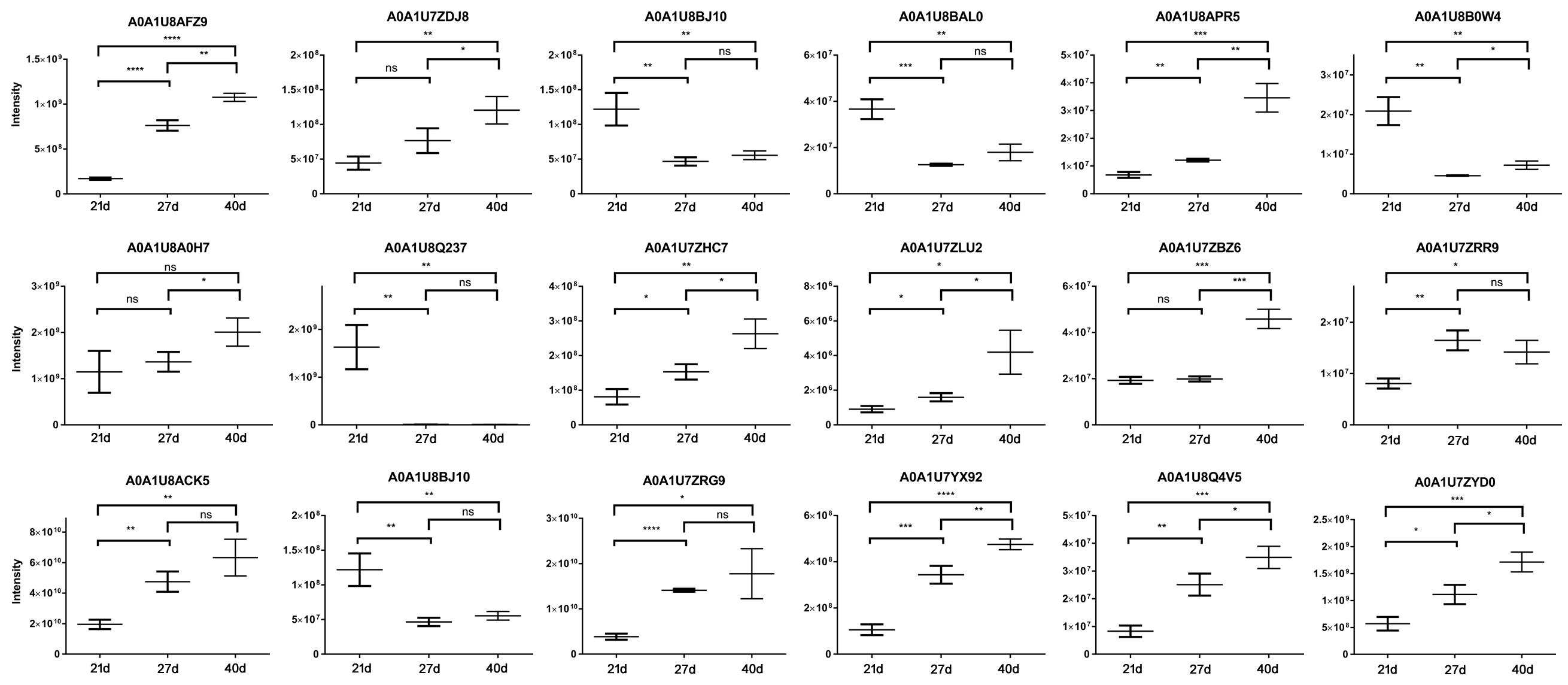

Supplement: Supplementary Figure 5 — Parallel reaction monitoring quantitative verification of eighteen proteins from tandem mass tags (TMT) proteomics data. Asterisks indicate statistically significant differences (***p < 0.001; **p < 0.01; *p < 0.05) between lotus embryos at different dehydration stages. [file Image_5.TIF]

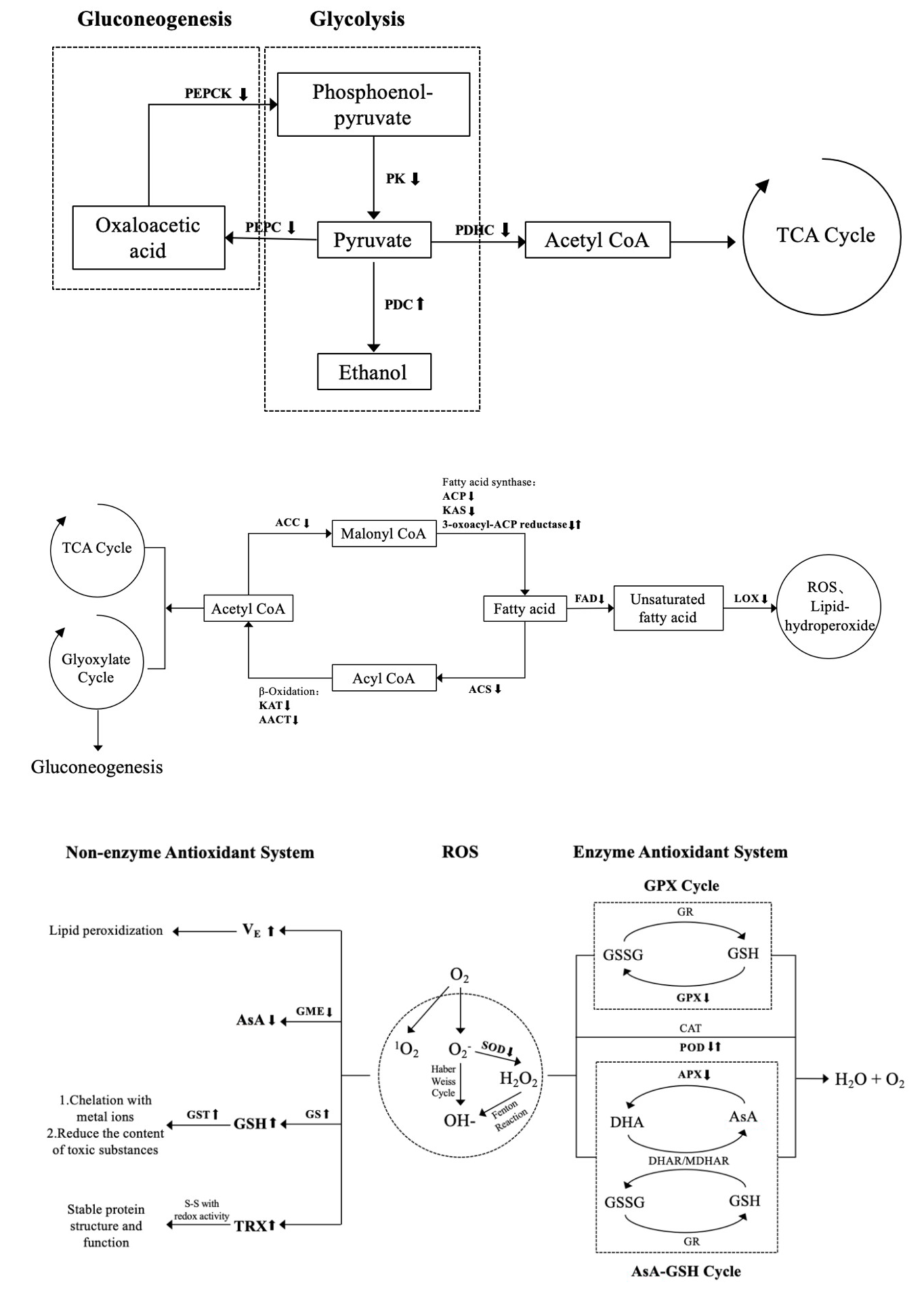

Supplement: Supplementary Figure 6 — Carbohydrates, energy, and antioxidation system regulation model of key DEPs from lotus embryo during seed dehydration maturity. [file Image_6.TIF]
