# Supplementary material for: TMT-Based Quantitative Proteomic Analysis Reveals the Physiological Regulatory Networks of Embryo Dehydration Protection in Lotus (Nelumbo nucifera)
Source: Front Plant Sci. 2021 Dec 17;12:792057. doi: 10.3389/fpls.2021.792057 (PMC8718645; doi:10.3389/fpls.2021.792057)
Supplement: Supplementary Table 3 — Functional classification of key DEPs from lotus embryo during dehydration maturity. [file Table_3.DOCX]

Supplementary Table 3. Functional classification of key DEPs from lotus embryo during dehydration maturity

| **Uniport Accession ID** | **Protein description** | **Fold change** | | |
| --- | --- | --- | --- | --- |
|  |  | **27/21** | **40/21** | **40/27** |
| **1. Carbohydrate and energy metabolism** | | | | |
| *Glycolsis/ Gluconeogenesis* | | | | |
| A0A1U8A1U3 | probable fructokinase 7 | ns | 1.526 | ns |
| A0A1U7ZX88 | ATP-dependent 6-phosphofructokinase | 1.900 | ns | 0.520 |
| A0A1U8AFZ9 | pyruvate decarboxylase 2 | 2.286 | 2.416 | ns |
| A0A1U8BCI6 | pyruvate decarboxylase 1-like | 1.513 | 1.581 | ns |
| A0A1U7ZA61 | alcohol dehydrogenase-like | 1.959 | 2.171 | n**s** |
| A0A1U8BCK9 | pyruvate, phosphate dikinase | 1.969 | 1.184 | 0.601 |
| A0A1U8BD39 | fructose-bisphosphate aldolase | 1.557 | 1.608 | ns |
| A0A1U8AIP2 | enolase 1 | ns | 0.631 | ns |
| A0A1U7YTA5 | isocitrate lyase | 0.482 | 0.413 | ns |
| A0A1U8BJ10 | phosphoenolpyruvate carboxylase | 0.650 | 0.548 | ns |
| A0A1U8BCN0 | phosphoenolpyruvate carboxylase | ns | 0.626 | ns |
| A0A1U8B3U2 | phosphoenolpyruvate carboxykinase | 0.450 | 0.393 | ns |
| A0A1U8AGB9 | pyruvate kinase | 0.277 | 0.251 | ns |
| A0A1U8BD18 | pyruvate kinase | 0.368 | 0.370 | ns |
| A0A1U8B3F0 | pyruvate kinase | 0.478 | 0.429 | ns |
| A0A1U8AKE4 | probable fructokinase-1 | ns | 0.656 | ns |
| *Pentose phosphate pathway* | | | | |
| A0A1U7ZDJ8 | glucose-6-phosphate dehydrogenase | 1.517 | 1.553 | ns |
| A0A1U7ZD76 | probable 6-phosphogluconolactonase | 1.968 | 1.761 | ns |
| *TCA cycle* | | | | |
| A0A1U8BAL0 | citrate synthase | 0.652 | 0.576 | ns |
| A0A1U7ZIW8 | pyruvate dehydrogenase | 0.365 | 0.345 | ns |
| A0A1U8AYI3 | pyruvate dehydrogenase | 0.495 | 0.460 | ns |
| A0A1U8A4V0 | pyruvate dehydrogenase complex | ns | 0.638 | ns |
| A0A1U8A3G1 | dihydrolipoyl dehydrogenase 2 | 0.641 | 0.627 | ns |
| *RFOs metabolism* | | | | |
| A0A1U8APY8 | UDP-glucose 4-epimerase GEPI48 | 1.998 | 2.047 | ns |
| A0A1U8Q8U3 | bifunctional UDP-glucose 4-epimerase | 1.939 | 2.062 | ns |
| A0A1U8B9X9 | inositol 3-α-galactosyltransferase | 2.580 | 2.377 | ns |
| A0A1U8APR5 | sucrose synthase | 2.069 | 2.302 | ns |
| A0A1U8A842 | UDP-glucose 6-dehydrogenase | 0.373 | 0.330 | ns |
| **2. Redox homeostasis** | | | | |
| A0A1U7ZUJ5 | glutathione synthetase | 1.585 | 1.788 | 1.788 |
| A0A1U8A0H7 | glutathione S-transferase-like | 1.553 | 1.570 | ns |
| A0A1U7YZC7 | microsomal glutathione S-transferase 3-like | 1.783 | 2.037 | ns |
| A0A1U8B339 | glutathione peroxidase | 0.654 | 0.633 | ns |
| A0A1U7ZEG8 | peroxidase | 1.543 | 1.514 | ns |
| A0A1U8AFN4 | peroxidase | 1.722 | 1.775 | ns |
| A0A1U8AT08 | L-ascorbate peroxidase 3 | ns | 0.611 | ns |
| A0A1U8Q237 | L-ascorbate peroxidase 2 | 0.202 | 0.188 | ns |
| A0A1U7ZF27 | 2-Cys peroxiredoxin BAS1 | 2.300 | 2.416 | ns |
| A0A1U8BMU4 | peroxidase | 0.379 | 0.402 | ns |
| A0A1U8B6F0 | 1-Cys peroxiredoxin-like | ns | 0.612 | ns |
| A0A1U7ZHC7 | thioredoxin-like protein CXXS1 | 1.639 | 1.908 | ns |
| A0A1U8B4Q2 | superoxide dismutase [Fe] 3 | 0.616 | 0.561 | ns |
| **3. Stress/Defense** | | | | |
| A0A1U8Q3Y8 | small heat shock protein | 2.438 | 2.788 | ns |
| A0A1U7ZRD1 | heat shock 70 kDa protein 8 | 1.784 | 1.725 | ns |
| A0A1U8AH10 | 16.9 kDa class I heat shock protein 1-like | 2.470 | 2.488 | ns |
| A0A1U8AV83 | 17.8 kDa class I heat shock protein-like | 2.116 | 1.762 | ns |
| A0A1U8B6M1 | 17.8 kDa class I heat shock protein-like | 2.185 | 1.601 | ns |
| A0A1U8B7B0 | 17.5 kDa class I heat shock protein-like | 1.555 | ns | ns |
| A0A1U7YQH | 18.2 kDa class I heat shock protein-like | 1.662 | 1.607 | ns |
| A0A1U8B8R0 | 17.3 kDa class II heat shock protein-like | 2.366 | 1.952 | ns |
| A0A1U8B630 | 15.7 kDa heat shock protein | 0.631 | 0.600 | ns |
| A0A1U7ZVY9 | heat shock cognate 70 kDa protein 2-like | 0.640 | 0.598 | ns |
| A0A1U8AZG4 | heat shock 70 kDa protein-like | ns | 0.652 | ns |
| A0A1U8A4L1 | heat shock protein 83 | ns | 0.636 | ns |
| A0A1U8BJF5 | heat shock protein 83-like | ns | 0.577 | ns |
| A1E2A9 | 17.1 kDa class II heat shock protein | ns | 0.619 | ns |
| A0A1U7ZPG1 | dnaJ homolog subfamily B member 13-like | ns | 0.549 | ns |
| A0A1U7ZYB6 | dnaJ protein homolog 2-like isoform X1 | ns | 0.622 | ns |
| A0A1U8AKE7 | dnaJ protein homolog | ns | 0.636 | ns |
| A0A1U7ZRR9 | dehydrin Rab18-like isoform X1 | 14.646 | ns | 0.082 |
| A0A1U8AH73 | late embryogenesis abundant protein Lea14-A | 1.893 | 2.097 | ns |
| A0A1U8BK19 | late embryogenesis abundant protein D-34-like | 2.121 | 2.204 | ns |
| A0A1U7ZRG9 | late embryogenesis abundant protein D-34 | 2.203 | 2.338 | ns |
| A0A1U8BJB0 | late embryogenesis abundant protein D-34-like | 2.230 | 2.304 | ns |
| A0A1U8A468 | late embryogenesis abundant protein D-34-like | 1.837 | 1.950 | ns |
| A0A1U8ACK | late embryogenesis abundant EMB564-like | 2.173 | 2.241 | ns |
| A0A1U8A6G | 11 kDa late embryogenesis abundant protein | 2.604 | 3.021 | ns |
| A0A1U8AZH5 | universal stress protein PHOS34 | 1.579 | ns | ns |
| A0A1U7ZBZ6 | protein-L-isoaspartate O-methyltransferase | ns | 1.508 | ns |
| A0A1U8AGU3 | probable aquaporin PIP2-8 | 1.604 | 1.602 | ns |
| A0A1U8AYP2 | protein early responsive to dehydration 15 | 0.438 | 0.419 | ns |
| A0A1UYZX0 | protein early responsive to dehydration 15-like | 0.430 | 0.428 | ns |
| **4. Protein modification and degradation** | | | | |
| A0A1U8BCK5 | 50S ribosomal protein L34 | 1.675 | 1.732 | ns |
| A0A1U8AZM9 | 60S ribosomal protein L14-2-like | 2.814 | ns | 0.385 |
| D3WCA0 | 30S ribosomal protein S16 | 0.623 | 0.620 | ns |
| A0A1U7YW34 | histone deacetylase HDT1-like isoform X1 | 2.003 | 2.277 | ns |
| A0A1U8Q4V5 | histone deacetylase 2 isoform X1 | 2.110 | 1.858 | ns |
| A0A1U8BLQ1 | histone deacetylase HDT1-like | 1.549 | 1.820 | ns |
| A0A1U7YX92 | cysteine proteinase inhibitor | 2.215 | 2.383 | ns |
| A0A1U8Q3X2 | cysteine proteinase inhibitor | ns | 1.536 | ns |
| A0A1U7Z8B4 | probable cysteine protease RD19D | 0.600 | 0.607 | ns |
| A0A1U8BBP5 | subtilisin-like protease SBT 2.6 | 0.496 | 0.442 | ns |
| A0A1U8AYU8 | subtilisin-like protease SBT 1.7 | 0.507 | 0.454 | ns |
| A0A1U8BC75 | subtilisin-like protease SBT 1.5 | 0.632 | 0.651 | ns |
| **5. Response to ABA signaling** | | | | |
| A0A1U8B646 | abscisic acid-insensitive 5-like isoform X1 | ns | 1.906 | ns |
| A0A1U7ZTI7 | abscisic acid-insensitive 5-like protein 2 | ns | 0.642 | ns |
| A0A1U7YYQ4 | abscisic acid-insensitive 5-like protein | ns | 0.638 | ns |
| **6. DNA repair** | | | | |
| A0A1U7ZYD0 | DNA damage repair protein | 1.730 | 1.676 | ns |
| A0A1U7ZKY2 | HMG B protein 1-like isoform X1 | 2.147 | 2.775 | ns |
| A0A1U8ATJ2 | HMG1/2-like protein | 2.008 | 2.846 | ns |
| A0A1U7Z4F5 | HMG1/2-like protein | ns | 1.849 | ns |
| A0A1U7Z5K0 | histone H2B.9-like | 3.371 | 3.980 | ns |
| A0A1U8AWH0 | late histone H2B.2.2-like | ns | 1.522 | ns |

‘ns’ means the abundance change of the protein was not significant.
