# Supplementary material for: TMT-Based Quantitative Proteomic Analysis Reveals the Physiological Regulatory Networks of Embryo Dehydration Protection in Lotus (Nelumbo nucifera)
Source: Front Plant Sci. 2021 Dec 17;12:792057. doi: 10.3389/fpls.2021.792057 (PMC8718645; doi:10.3389/fpls.2021.792057)
Supplement: Supplementary Table 4 — Correlation analysis of stress physiological indices of lotus embryo during dehydration maturity. [file Table_4.DOCX]

Supplementary Table 4. Correlation analysis of stress physiological indices of lotus embryo during dehydration maturity

|  | RWC | REC | MDA | H_2_O_2_ | O_2_^·-^ | OH· | SOD | POD | CAT | AsA | GSH | V_E_ |
| --- | --- | --- | --- | --- | --- | --- | --- | --- | --- | --- | --- | --- |
| RWC | 1 | **-.877**** | **-.844**** | **-.960**** | **.895**** | **.929**** | 0.067 | .783* | 0.44 | **.938**** | **-.986**** | **-.807*** |
| REC | — | 1 | **.925**** | **.945**** | -0.656 | **-.844**** | 0.053 | -0.595 | -0.392 | **-.929**** | **.932**** | **.726*** |
| MDA | — | — | 1 | **.958**** | **-.766*** | **-.822*** | -0.273 | -0.61 | -0.209 | **-.783*** | **.879**** | **.847**** |
| H_2_O_2_ | — | — | — | 1 | **-.856**** | **-.928**** | -0.173 | **-.748*** | -0.319 | **-.907**** | **.970**** | **.870**** |
| O_2_^·-^ | — | — | — | — | 1 | **.836**** | 0.441 | **.793*** | 0.155 | 0.693 | **-.832*** | **-.880**** |
| OH· | — | — | — | — | — | 1 | 0.199 | **.931**** | 0.171 | **.897**** | **-.895**** | **-.907**** |
| SOD | — | — | — | — | — | — | 1 | 0.36 | **-.730*** | -0.202 | 0.017 | -0.573 |
| POD | — | — | — | — | — | — | — | 1 | -0.067 | **.709*** | -0.697 | **-.883**** |
| CAT | — | — | — | — | — | — | — | — | 1 | 0.527 | -0.506 | 0.151 |
| AsA | — | — | — | — | — | — | — | — | — | 1 | **-.958**** | -0.674 |
| GSH | — | — | — | — | — | — | — | — | — | — | 1 | **.758*** |
| V_E_ | — | — | — | — | — | — | — | — | — | — | — | 1 |
